# Chemogenetic manipulation of learning‐tagged neurons is sufficient to rescue progressive memory deficits in a mouse model of Alzheimer's disease

**DOI:** 10.1002/alz.70953

**Published:** 2025-11-27

**Authors:** Stefano Guglielmo, Niccolò Di Nardo, Marco Scantamburlo, Marco Mainardi, Michel C. van den Oever, Nicola Origlia

**Affiliations:** ^1^ BIO@SNS Laboratory Scuola Normale Superiore Pisa Italy; ^2^ Institute of Neuroscience National Research Council Pisa Italy; ^3^ Department of Biomedical Sciences University of Padova Padova Italy; ^4^ Department of Molecular and Cellular Neurobiology Center for Neurogenomics and Cognitive Research (CNCR), Amsterdam Neuroscience Vrije Universiteit Amsterdam Amsterdam the Netherlands

**Keywords:** Alzheimer's disease, chemogenetic reactivation, engram, entorhinal‐hippocampal network, episodic memory

## Abstract

**INTRODUCTION:**

Alzheimer's disease (AD) primarily affects episodic memory, which relies on the medial temporal lobe, including the hippocampus and lateral entorhinal cortex (LEC). However, it remains unclear whether memory deficits in AD reflect disrupted encoding of new experiences or impaired retrieval of previously stored information.

**METHODS:**

APPJ20 transgenic mice were used to investigate memory deficits. Neuronal populations activated during the learning phase of associative and non‐associative tasks were tagged to express the excitatory chemogenetic receptor hM3Dq. Chemogenetic activation of these tagged neurons was performed during the recall phase of the tasks.

**RESULTS:**

Chemogenetic reactivation of LEC or dentate gyrus (DG) learning‐tagged neurons rescued memory performance in associative and non‐associative tasks, respectively. Neuronal activation, assessed using c‐Fos as a marker, revealed a specific deficit in the reactivation of neurons recruited during learning.

**DISCUSSION:**

Chemogenetic reactivation of neuronal ensembles in the LEC and DG restored memory performance, suggesting that memory deficits in APPJ20 mice are associated with a failure in the endogenous reactivation of learning‐relevant neurons.

**Highlights:**

- APPJ20 mice exhibited early entorhinal synaptic dysfunction and impaired episodic‐like memory retrieval. At a later stage, hippocampal synaptic function became impaired, leading to altered non‐associative memory performance.
- The analysis of neuronal activation using c‐Fos revealed a specific impairment of the subpopulation recruited during memory encoding.
- Chemogenetic reactivation of LEC learning‐tagged neurons rescued associative memory performance in 2‐month‐old APPJ20 mice, while promoting dendritic spine maturation and stabilization in LEC neurons.
- Chemogenetic reactivation of DG learning‐tagged neurons in 6‐month‐old APPJ20 mice restored non‐associative memory retrieval.
- This study supports the hypothesis that during AD progression, memory is encoded but not accessible through natural cues alone.

## 1 BACKGROUND

Alzheimer's disease (AD) is a complex, multifactorial, neurodegenerative disorder characterized by the presence of extracellular amyloid beta (Aβ) plaques and intracellular neurofibrillary tangles of hyperphosphorylated tau protein.^1^ AD represents the leading cause of dementia and cognitive decline, with early stages marked by progressive episodic memory deterioration. Episodic memory is defined as a declarative memory that contains information specific to the time and place of acquisition,^2^ and it is highly dependent on the medial temporal lobe system, which includes the hippocampus and entorhinal cortex (EC).^3^, ^4^ Recently, numerous studies have demonstrated the pivotal role of the lateral part of the entorhinal cortex (LEC) in contextual and temporal information processing.^5^, ^6^ Notably, the LEC is involved in encoding associations between objects, places, and contexts, which are fundamental components of episodic memory.^7^, ^8^, ^9^ This population activity in the EC is directed to the hippocampus, where these signals are integrated and stored as unique ensemble activity, creating a cohesive representation of experience.^10^ The superficial layers of the EC are particularly affected in the early stages of AD, leading to a significant loss of neurons in EC layer II, which provides the major input to the granule cells of the dentate gyrus (DG).^11^, ^12^ Aβ accumulation has been considered the molecular driver of AD that initiates a cascade of events that ultimately lead to synaptic dysfunction, neuronal loss, and cognitive decline.^13^ Aggregated forms of Aβ exhibit prion‐like behavior due to their capacity for seeding, spreading, and propagating, supporting the hypothesis that AD may progress through anatomically and functionally connected brain regions.^14^, ^15^ Using a transgenic mouse model that overexpresses human amyloid precursor protein (APP) with two mutations linked to familial AD (the Swedish and Indiana mutations, the APPJ20 strain),^16^ it has already been demonstrated that EC synaptic function and dendritic spine morphology are affected prior to any detectable plaque accumulation, driving an impairment of episodic‐like memory retrieval evaluated using the object‐place‐context recognition task (OPCRT). Subsequently, synaptic function becomes affected in the EC main target region, the hippocampus, leading to the loss of non‐associative memory as assessed by the novel object recognition task.^17^ However, it remains uncertain whether the episodic memory impairment associated with AD arises primarily from disruptions in the encoding and consolidation processes necessary for forming new memories or from specific deficits in the mechanisms required to retrieve previously stored information. Studies suggest that memory failure in early AD models primarily reflects an impairment in the retrieval of information.^18^, ^19^ Recently, using a dual viral system based on targeted recombination in active populations (TRAP) technology,^20^ we demonstrated that neuronal ensembles within the LEC supported the encoding and recall of object‐place‐context (OPC) associations. This finding suggests the presence of a LEC engram for such memory traces.^21^ To determine whether the episodic memory retrieval deficit in transgenic APP mice arose from a defect in memory encoding or recall, we expressed the excitatory chemogenetic receptor hM3Dq in the subpopulation of LEC neurons activated during the learning phase of the OPCRT. This approach allowed us to assess whether chemogenetic activation of this specific LEC neuronal population was sufficient to rescue memory performance in young adults, 2‐month‐old APP mice. Furthermore, we hypothesize that the impairment of novel object recognition memory in 6‐month‐old APP mice results from the progression of amyloid pathology to the hippocampus and could be rescued using the same chemogenetic approach in the DG.

## 2 METHODS

### 2.1 Animals

All experimental procedures involving animals followed the guidelines defined by European legislation (Directive 2010/63/EU) and Italian Legislation (LD no. 26/2014). The Organism Responsible for Animal Welfare (OPBA) of the National Research Council of Italy (CNR) Institute of Neuroscience in Pisa and the Italian Ministry of Health approved the study protocol (authorization no. 16/2022‐PR). PDGF‐APPSw, Ind (line J20) transgenic mice overexpressing an alternatively spliced human APP minigene that encodes hAPP695, hAPP751, and hAPP770 bearing mutations linked to familial AD (V717F, K670N/M671L) have been used (Charles River, Italy).^20^ Heterozygous J20‐hAPP male mice were bred with C57BL/6J female mice to maintain the hAPP transgene as heterozygous. Throughout this paper, the APPJ20 mouse model will be referred to as the APP mouse model for consistency. The resulting litters, which included both male and female heterozygous APP mice along with their non‐transgenic C57BL/6J (Charles River, Italy) littermates, were used for experiments after genotype confirmation. Detailed information on the number of animals per sex for each experiment is provided in the Supporting Information (Tables S1–S10). Mice were housed in conventional cages (365 × 207 × 140 mm, two to three animals per cage) with nesting material on a 12‐h light/dark cycle with food and water available ad libitum. Behavioral experiments were performed on 2‐ and 6‐month‐old mice during the light phase, and mice were randomly assigned to experimental groups. To control for order and cage effects, each cage contained a mixture of mice from the experimental and control groups. The number of animals used in each experiment is provided in the figure legends.

**RESEARCH IN CONTEXT**

1. **Systematic review**: We reviewed literature on memory impairments in Alzheimer's disease (AD) using PubMed and Google Scholar. Early dysfunction of the lateral entorhinal cortex (LEC) contributes to episodic memory decline, while hippocampal involvement emerges later, indicating distinct phases. However, it remains unclear whether these deficits reflect impaired encoding or disrupted retrieval of stored information.
2. **Interpretation**: Our findings suggest that memory deficits in APPJ20 mice result from failed reactivation of learning‐relevant ensembles in the LEC and dentate gyrus (DG). Chemogenetic reactivation restored memory, indicating intact encoding of associative and non‐associative memories at both early and later stages.
3. **Future directions**: This study emphasized that memory retrieval deficits in AD are linked to impaired reactivation of neurons recruited during encoding, rather than disruptions of encoding itself. The ability to recover inaccessible memory traces through artificial stimulation opens possibilities for future therapeutic strategies aimed at restoring memory function in AD patients.

### 2.2 Adeno‐associated virus (AAV) vectors and stereotaxic injections

The following AAV vectors were used for the chemogenetic manipulation: AAV5‐Fos::CreER^T2^ (titer 1.2 × 10^13^), AAV5‐hSyn::DIO‐hM3Dq‐mCherry, and AAV‐hSyn::DIO‐mCherry (titers: 5.0 to 6.0 × 10^12^).

For stereotaxic injections, 2‐ or 6‐month‐old mice were deeply anesthetized using an intraperitoneal (i.p.) injection of Zoletil 100 (zolazepam and tiletamine, 1:1, 40 mg/kg; Laboratoire Virbac) and Xilor (xylazine 2%, 10 mg/kg; Bio98). Lidocaine (2%) was topically applied to the skull to provide local analgesia. A bilateral craniotomy was performed at the stereotaxic coordinates targeting the LEC (anteroposterior [AP] −4.0 mm, mediolateral [ML] ± 4.0 mm) and the DG (AP −2.0 mm, ML ± 1.0 mm, dorsoventral −1.72 mm from the brain surface). For the LEC, a glass pipette was lowered from the brain's surface at an 11° angle until a slight bend in the pipette indicated contact with the dura. The pipette was retracted 0.1 mm, and 250 nL of a virus mixture of AAV5‐FosCreER^T2^ and Cre‐dependent AAV (ratio 1:500, AAV5‐FosCreER^T2^ at a final titer of 2.43 × 10^10^) was injected. Each hemisphere received the virus mixture at a flow rate of 0.1 µL/min, followed by an additional 5 min to allow diffusion of the virus. Animals remained in their home cage for 3 weeks until the start of behavioral experiments.

### 2.3 Behavioral tasks

The test environment was composed of two square boxes (length 40 cm, width 40 cm, height 40 cm) with different visual cues on the walls to provide distinct contexts. The wall and floor of the environment were cleaned with 30% alcohol before each trial. The objects were household items of approximately the same size as the mouse and varying in color, shape, and texture. To avoid odor cues, new identical copies of each object were used for each trial, and objects were cleaned with 30% alcohol after each trial.

Before the behavioral tests, mice were habituated to the experimenter and room by extensive handling for 2 weeks. For the OPCRT, mice were allowed to explore the first context for 5 min before the beginning of the sample phases. Subsequently, mice were presented with two distinct novel objects in Context A during the first sample trial. After this trial, the mice were removed from the box and placed in a holding cage for a 1‐min intertrial interval (ITI) while the box was cleaned. In the second sample trial, the same pair of objects was presented in Context B, but their positions were reversed relative to the first trial. During the test trial, mice were exposed to a pair of identical objects from the sample trials in the same context and positions as in the first sample trial. Both the sample phases and the test phase were conducted for a duration of 10 min each. The test trial was performed 12 h after the sample‐trial presentation. The novel object recognition test (ORT) was performed following the same procedure described for the OPCRT. In this case, mice were exposed to two identical objects during the sample trials, and 12 h later the test trial consisted of one familiar and one novel object. The duration of exploration in each phase was the same as in the OPCRT. In all the experiments, mice were judged to be exploring an object when it was in proximity with their nose directed toward it. Exploration time was not counted when the mouse's nose was directed away from the object. To ensure reliability, the same separate observer re‐scored all the videos in a blind fashion for each task, and these scores consistently differed by less than 10% from those of the experimenter. For each task, observation scores were converted to discrimination indices (discrimination index [DI] = [time at novel − time at familiar [/[time at novel + time at familiar]) to evaluate the extent to which mice explored novel versus familiar objects. The scoring of the videos was performed using Chronotate,^22^ a software designed for precise manual analysis of behavior during experimental trials. The marker output files generated by Chronotate were processed using a custom Python script, which allowed for the quantification of the total exploration time as well as the total number of interactions with both the novel and familiar objects. Behavioral videos were recorded using an AUKEY 1080p full high‐definition webcam and were subsequently analyzed offline. Different body regions of the mouse – namely, the nose, two ears, back, middle portion, and tip of the tail – were labeled using the open‐source tool DeepLabCut^23^ for marker‐less pose estimation. This animal tracking enabled the automated calculation of various motor parameters, facilitating a more detailed analysis of the animal's behavior.

### 2.4 Pharmacological treatment

4‐hydroxytamoxifen (4OH‐TAM) (H6278, Sigma‐Aldrich) was prepared for injection by dissolving it in saline. A stock solution of 50 mg/mL 4OH‐TAM in DMSO (D8418, Sigma‐Aldrich) was initially prepared and stored at −20°C. On the day of the experiment, the final working solution of 2.5 mg/mL 4OH‐TAM was achieved in two stages: first, by diluting the stock 1:10 in saline that contained 2% Tween80 (P1754, Sigma‐Aldrich) and then adding a volume of saline. This final solution consisted of 2.5 mg/mL 4OH‐TAM, 5% DMSO, and 1% Tween80 in saline. Mice were given an i.p. injection of 4OH‐TAM at a dose of 25 mg per kg body weight, 4 h prior to the sample trials.

Clozapine N‐oxide hydrochloride (CNO; Catalog No.: 34233–7, Merck) was dissolved in sterile saline for injection. For the behavioral experiments, mice were administered 3 mg/kg (i.p.) of CNO 30 min before each test phase.

### 2.5 Immunohistochemistry

Mice were deeply anesthetized with urethane (Merck, 20% solution, 0.1 mL/100 g body weight) and perfused intracardially with phosphate‐buffered saline (PBS) (pH 7.4), followed by 4% paraformaldehyde (PFA) in PBS (pH 7.4). The brains were carefully removed, post‐fixed overnight in 4% PFA (w/v), and transferred to a 30% sucrose solution (w/v) in PBS for dehydration and cryoprotection. Coronal sections, 50 µm thick, were prepared using a Leica freezing microtome, and the free‐floating slices were subsequently processed for immunofluorescence analysis. Following the selection of appropriate brain slices, the sections were incubated for 2 h at room temperature in a blocking solution containing 5% bovine serum albumin (BSA) (w/v) and 0.5% Triton X‐100 (v/v) in PBS. They were then incubated overnight at 4°C with a monoclonal anti‐cFos antibody (Catalog No.: 226 008, Synaptic Systems) diluted 1:1000 and a monoclonal anti‐mCherry antibody (Catalog No.: M11217, Thermo Fisher Scientific) diluted 1:1000, both prepared in PBS with 1% BSA (w/v) and 0.1% Triton X‐100 (v/v). Afterward, the slices were washed three times for 5 min with PBS at room temperature and incubated for 2 h at room temperature with Alexa Fluor 488‐conjugated secondary antibody (Catalog No.: 711‐545‐152, Jackson Immunoresearch) and Alexa Fluor 568‐conjugated secondary antibody (Catalog No.: A11077, Thermo Fisher Scientific), both diluted 1:500 in the same solution used for primary antibody incubation. The sections were then washed three times with PBS, mounted onto slides, air‐dried, and cover‐slipped using Fluoromount aqueous mounting medium (Catalog No.: F4680, Merck).

### 2.6 Image acquisition

Fluorescence images were captured using a Leica Stellaris 8 confocal microscope equipped with 10x/0.3 dry and 20x/0.75 dry objectives. Each brain slice was imaged as a tiled acquisition spanning four to six z‐planes. Images were acquired at a scan speed of 600 Hz with a resolution of 2048 × 2048 pixels. For dendritic spine imaging, a Zeiss LSM 800 confocal laser scanning microscope equipped with an AiryScan detector and a 63x/1.40 oil immersion objective was used. Images were acquired at 16‐bit depth using AiryScan imaging, a super‐resolution microscopy technique. Z‐stacks were captured with a step size of 0.15 µm, spanning the entire volume of the selected dendritic region. Raw images were processed using AiryScan image reconstruction in ZEN software.

### 2.7 Spine analysis

For spine analysis, eight pyramidal neurons were selected per mouse from layer II/III of the LEC. From each neuron, two 20‐µm segments of second‐order apical dendrites were randomly chosen for detailed spine morphology analysis using the Filament Tracer module in Imaris software (version 7.4.2). In the Filament Tracer's Surpass mode, the Autopath algorithm was used to trace dendrites and spines. Reconstruction was conducted using a semi‐automatic approach, with dendrite diameters constrained to 1 to 5 µm and a minimum spine diameter of 0.4 µm. Automated thresholds were initially applied to identify spine seed points and enable surface rendering; however, threshold values were manually adjusted for each dendrite segment to ensure accuracy, adding true spines or removing artifacts as necessary. The morphological properties of dendrites and spines were exported in an .xls table using a custom‐made Xtension script in MATLAB. A data refinement procedure was conducted to enhance the reliability of the dataset by removing spines generated during automated reconstruction that either lacked a spine neck or were protrusions incorrectly identified as spines.

Spine morphology was then analyzed according to its width, length, and length/width ratio (LWR), with the criteria from Barón‐Mendoza et al. (2021)^24^ modified as follows:

1. Filopodia: length > 2 µm, LWR > 1 spine head max diameter ≤ 0.6 µm
2. Long thin: 1 µm < length ≤ 2 µm, spine head max diameter ≤ 0.6 µm
3. Thin: length ≤ 1 µm, spine head max diameter ≤ 0.6 µm
4. Stubby: LWR ≤ 1
5. Mushroom: spine head max diameter ≥ 0.6 µm

Spine area and volume were computed following the approach described in Imaris software. Specifically, the surface area (or volume) of the hemisphere at the spine attachment point was subtracted from the sum of the surface area (or volume) of the spine frustum and the terminal hemisphere.

Data processing and spine classification were performed in Python using a custom‐made script.

### 2.8 Cell counting

To analyze the expression pattern of c‐Fos within the medial temporal lobe, hippocampal and entorhinal subregions were manually outlined using ImageJ. The outlines were guided by the mouse brain atlas.^25^ The detection of c‐Fos^+^ cells was achieved using a custom Python script that used the pre‐trained model “2D_Versatile_Fluo” from StarDist,^26^ a deep learning‐based method for cell and nuclei detection and segmentation. The output of the segmentation process was refined by applying a semi‐automated threshold for intensity and area. These parameters were adjusted manually for individual images when necessary to account for inaccuracies. The density of c‐Fos^+^ cells (c‐Fos^+^/mm^2^) was averaged over four to five sections per animal. The same method was used to count the DAPI^+^ cells. For the mCherry+ cell detection, cell counting was manually performed in ImageJ and the mCherry^+^ cell density (mCherry^+^/mm^2^) was averaged over four to five sections per animal. The number of c‐Fos⁺/mCherry⁺ cells per section was manually counted in ImageJ. *Z*‐planes were examined to ensure accurate co‐localization of the c‐Fos and mCherry signals within the same cell. The percentage of reactivation within the tagged neuronal population was calculated as ([c‐Fos^+^ mCherry^+^]/[mCherry+]) × 100. To assess the level of reactivation, the proportion of overlap with DAPI⁺ was calculated as (c‐Fos⁺mCherry⁺)/DAPI⁺. The expected chance level of overlap was determined as (c‐Fos⁺/DAPI⁺) × (mCherry⁺/DAPI⁺), and this calculation was performed for each slice. Overlap/chance was then computed by dividing the overlap/DAPI value by the chance value for each slice. Overlap/chance was averaged across all slices for each mouse, generating a single average value used for statistical analysis.

For single‐cell mean fluorescence intensity analysis, the mask generated during the c‐Fos⁺ cell detection segmentation was imported into ImageJ. This mask was then used to select the population of c‐Fos⁺ cells. ImageJ was used to extract and average the mean intensity for each c‐Fos⁺ cell. The average intensity per animal was calculated from four or five slices.

### 2.9 Whole‐cell patch‐clamp recordings

Whole‐cell patch‐clamp recordings were performed as previously described^21^ on visually identified EC neurons. Briefly, for acute LEC slice preparation, the head was submerged in ice‐cold, oxygenated cutting solution containing (in mM): Sucrose 248, HEPES 20, glucose 10, MgCl2 10, KCl 2, CaCl2 0.5, Na+ ascorbate 5, Na+ pyruvate 3, pH adjusted to 7.4 with NaOH, where the brain was quickly extracted from the skull. The brain was immersed in the same ice‐cold, oxygenated cutting solution, and 250‐µm‐thick brain sections were cut using a vibratome (Leica VT1200S, Weitzlar, Germany), then transferred to a recovery chamber containing oxygenated artificial cerebrospinal fluid (aCSF) composed of (in mM) NaCl 119, HEPES 10, glucose 10, NaHCO3 6.2, KCl 2.5, CaCl2 2, MgCl2 1.2 NaH2PO4 1, Na+ ascorbate 0.4, pH adjusted to 7.4 with NaOH, in which they were incubated at 32°C for 30 min. After moving the chamber to room temperature and allowing an additional 60 min, slices were transferred to the recording chamber, where recordings were performed under continuous perfusion with oxygenated aCSF at 32°C. Spontaneous excitatory postsynaptic currents (sEPSCs) were recorded in patch‐clamp mode. Borosilicate glass micropipettes were fabricated from 1B150F‐4 capillaries (WPI, Sarasota, FL, USA) using a PC‐100 vertical puller (Narishige International, London, UK) and had a 4‐ to 6‐MΩ resistance when filled with an internal solution containing (in mM) K+‐gluconate 145, HEPES 10, phosphocreatine 5, Mg2+‐ATP 2.5, MgCl2 2, Na+‐GTP 0.25, EGTA 0.1, plus biocytin (HB5035, Hello Bio, Dunshaughlin, Ireland) 0.2% w/v, pH adjusted to 7.35 with KOH. Neurons of the superficial layers of the LEC were approached under differential interference contrast illumination using a 63× immersion objective. After establishing a gigaseal, the patch was broken by applying negative pressure to achieve a whole‐cell configuration. A series resistance lower than 15 MΩ was considered acceptable and monitored constantly throughout the entire recording. At least 3 min were allowed for complete cytosol dialysis, then sEPSCs were recorded while holding the neuron at −70 mV, using a MultiClamp 700A amplifier and a Digidata 1550B card, controlled via Clampex 10 software (Molecular Devices, San Jose, CA, USA). Signal analysis was performed with the Clampfit 10 software (Molecular Devices) by employing the template‐based search and identification function, with a template match threshold of 4.

### 2.10 Statistical analysis

All data are presented as mean ± SEM. Statistical analyses were performed using GraphPad Prism 8 (GraphPad Software, San Diego, CA). Detailed statistical information for each experiment is provided in the corresponding figure legends. Briefly, data distribution was assessed using the Shapiro–Wilk test for normality. Depending on the experimental design, comparisons between groups were performed using one‐way or two‐way ANOVA followed by Sidak's multiple comparisons test, or an unpaired *t* test. For behavioral experiments, these tests were used to assess differences in discrimination indices and exploration parameters. One‐sample *t* tests were used to determine whether the mean DI within each group differed significantly from chance (hypothesized mea*n =* 0). Two‐way ANOVA followed by Sidak's multiple comparisons test was used to evaluate group differences in c‐Fos^+^ cell density and single‐cell mean fluorescence intensity. One‐way ANOVA followed by Sidak's multiple comparisons test was applied to assess differences in co‐localization signals and reactivation rates. For spine analysis, morphological features were compared using either a two‐way ANOVA with Sidak's multiple comparisons test or a two‐tailed unpaired *t* test, as appropriate. Principal component analysis (PCA)‐based clustering was performed in Python using the scikit‐learn library. A dataset comprising 16 morphological features was extracted from the 3D reconstruction of dendritic spines. Data were normalized using StandardScaler, and PCA was subsequently applied to reduce dimensionality. *K*‐means clustering was performed, with the optimal number of clusters determined using the elbow method.

## 3 RESULTS

### 3.1 Behavioral analysis and c‐Fos quantification showed no differences in OPC memory encoding in 2‐month‐old APP mice

It was previously shown that APP mice exhibited early impairment in OPC association retrieval at 2 months of age, preceding any detectable alterations in hippocampal function and non‐associative ORT.

**FIGURE 1:**
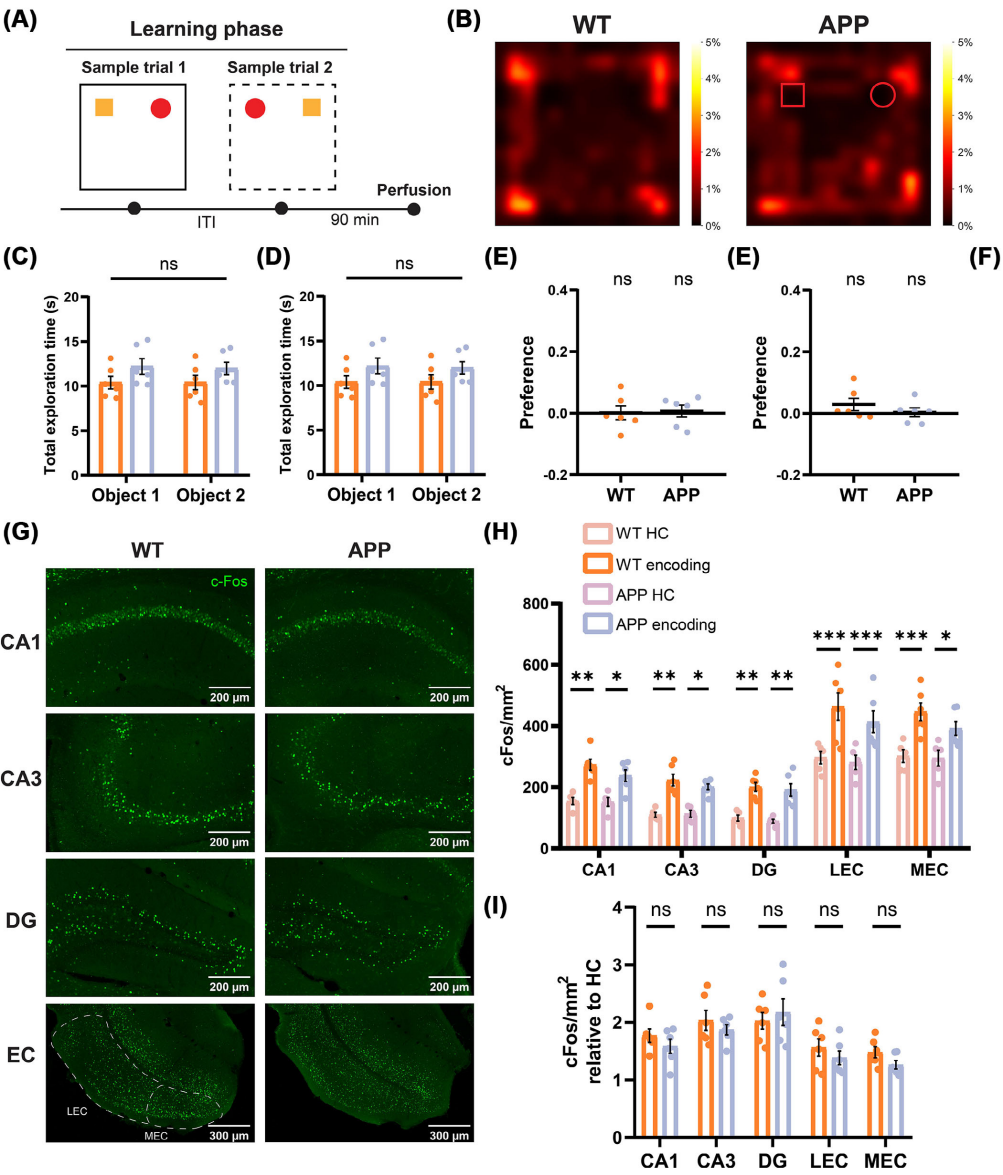
Behavioral analysis and c‐Fos quantification showed no differences in object‐place‐context memory encoding in 2‐month‐old APP mice. (A) Schematic representation of learning phase of object‐place‐context recognition task (OPCRT), with animal perfusion that was performed 90 min after the end of Sample trial 2. (B) Examples of heatmaps showing exploratory activity of mice (nose position) during Sample trial 1 of learning phase. The color bar represents the percentage of total time spent in each position. (C) Total exploration time during Sample trial 1 showed no differences between groups (two‐way ANOVA with Sidak's multiple comparisons test: WT Object 1 10.39 ± 0.70 *n =* 6 vs APP Object 1 12.19 ± 0.89 *n =* 6, ns *p =* 0.2223; WT Object 2 10.40 ± 0.80 *n =* 6 vs APP Object 2 11.97 ± 0.70 *n =* 6, ns *p =* 0.31). (D) Total exploration time during Sample trial 2 showed no differences between groups (two‐way ANOVA with Sidak's multiple comparisons test: WT Object 1 9.87 ± 0.90 *n =* 6 vs APP Object 1 11.03 ± 1.01 *n =* 6, ns *p =* 0.6293; WT Object 2 7.85 ± 0.67 *n =* 6 vs APP Object 2 11.01 ± 1.09 *n =* 6, ns *p =* 0.3583). (E) During Sample trial 1, mice showed no preference for either object, with exploration not differing from chance (WT 0.028 ± 0.02, *n =* 6, ns *p =* 0.9575, df = 5, *t =* 0.055; APP 0.003 ± 0.014, *n =* 6, ns *p =* 0.7437, df = 5, *t =* 0.3456; one‐sample *t* test) or between groups (WT vs APP, ns *p =* 0.8585, df = 10, *t =* 0.1829, two‐tailed unpaired *t* test). (F) During Sample trial 2, mice showed no preference for either object, with exploration not differing from chance (WT 0.001 ± 0.02, *n =* 6, ns *p =* 0.2168, df = 5, *t =* 1.413; APP 0.006 ± 0.02, *n =* 6, ns *p =* 0.8293, df = 5, *t =* 0.2271; one‐sample *t* test) or between groups (WT vs APP, ns *p =* 0.3302, df = 10, *t =* 1.023, two‐tailed unpaired *t* test). (G) Representative images showing c‐Fos expression (green) in the regions of interest for both groups, 90 min after the end of the learning phase. Scale bars are shown in the figures. (H) A significant difference in c‐Fos^+^ density was observed across all regions of interest between the HC and encoding conditions in both WT and APP groups (two‐way ANOVA with Sidak's multiple comparisons test: WT HC CA1 154.6 ± 11.69 *n =* 5 vs WT encoding CA1 273.4 ± 17.97 *n =* 6, **p = 0.0019; WT HC CA3 109.9 ± 7.75 *n =* 5 vs WT encoding CA3 223.6 ± 19.12 *n =* 6, **p = 0.0033; WT HC DG 99.22 ± 9.82 *n =* 5 vs WT encoding DG 200.9 ± 14.37 *n =* 6, *p = 0.0110; WT HC LEC 297 ± 20.68 *n =* 5 vs WT encoding LEC 463.4 ± 44.82 *n =* 6, ***p < 0.001; WT HC MEC 301.7 ± 20.75 *n =* 5 vs WT encoding MEC 446.1 ± 28.80 *n =* 6, ***p < 0.001; APP HC CA1 152 ± 14.45 *n =* 5 vs APP encoding CA1 237.8 ± 18.62 *n =* 6, *p = 0.0476; APP HC CA3 113.2 ± 11.11 *n =* 5 vs APP encoding CA3 201.1 ± 9.61 *n =* 6, *p = 0.0396; APP HC DG 88.83 ± 6.37 *n =* 5 vs APP encoding DG 190.9 ± 20.45 *n =* 6, *p = 0.0106; APP HC LEC 281.1 ± 23.48 *n =* 5 vs APP encoding LEC 413.3 ± 35.61 *n =* 6, *** *p <* 0.001; APP HC MEC 294.9 ± 25.93 *n =* 5 vs APP encoding MEC 392 ± 22.76 *n =* 6, *p = 0.0171). (I) No difference in c‐Fos protein expression following behavioral activation between groups across all regions of interest (two‐way ANOVA with Sidak's multiple comparisons test: WT encoding CA1 1.768 ± 0.116 *n =* 6 vs APP encoding CA1 1.584 ± 0.124 *n =* 6, ns *p =* 0.8877; WT encoding CA3 2.034 ± 0.144 *n =* 6 vs APP encoding CA3 1.87 ± 0.089 *n =* 6, ns *p =* 0.9282; WT encoding DG 2.025 ± 0.144 *n =* 6 vs APP encoding DG 2.174 ± 0.233 *n =* 6, ns *p =* 0.9501; WT encoding LEC 1.56 ± 0.15 *n =* 6 vs APP encoding LEC 1.383 ± 0.119 *n =* 6, ns *p =* 0.9022; WT encoding MEC 1.478 ± 0.095 *n =* 6 vs APP encoding MEC 1.262 ± 0.073 *n =* 6, ns *p =* 0.8016). c‐Fos density is normalized to the HC control condition. Data are presented as mean ± SEM. APP, amyloid precursor protein; df, degrees of freedom; DG, dentate gyrus; HC, home cage; LEC, lateral entorhinal cortex; MEC, medial entorhinal cortex; WT, wild type.

To determine whether the deficit in episodic memory retrieval is associated with altered behavioral performance during the encoding of episodic‐like memory, 2‐month‐old wild‐type (WT) and APP mice were subjected to the learning phase of the OPCRT to assess potential differences in behavioral and motor performance (Figure 1A,B). Both groups spent a similar amount of time exploring the objects (Figure 1C,D), with no preferences observed for either object 1 or object 2 in the two configurations (Figure 1E,F). Additionally, there were no significant differences in the total distance traveled within the arena during the two trials or in the number of interactions with the objects for both groups, indicating comparable exploratory and motor behaviors during the learning phase (Figure S1A–D). These findings suggest that during the encoding of OPC memory, no detectable alterations in behavioral performance were observed in young APP mice compared to WT.

To investigate whether the encoding of episodic‐like memory is associated with similar activation of the medial temporal lobe areas in the two groups, the number of neurons positive for the immediate early gene (IEG)‐encoded c‐Fos protein was evaluated 90 min after the learning phase of the behavioral task (Figure 1G). Interestingly, no significant difference in c‐Fos^+^ cell density was observed in the DG, CA1, CA3, or the two subdivisions of the EC (LEC and medial entorhinal cortex [MEC]) between the two groups. We also assessed baseline c‐Fos expression in home cage controls for both groups and found no significant differences in the number of c‐Fos+ cells between 2‐month‐old WT and age‐matched APP mice (Figure 1H). Furthermore, both groups exhibited comparable levels of c‐Fos staining following behavioral stimulation (Figure 1I and Figure S1E). To determine whether episodic‐like memory encoding affects not only the number of c‐Fos^+^ cells but also the relative amount of c‐Fos protein produced by individual neurons, the mean fluorescence intensity per cell was analyzed for each animal. However, no significant differences in c‐Fos fluorescence intensity were detected in the regions of interest (Figure S1F).

Overall, these data indicate that 2‐month‐old APP mice exhibit unaltered behavioral performance during the learning phase of the OPCRT and a normal activation pattern of the IEG c‐Fos within the medial temporal lobe system, suggesting that OPC association encoding might be unaffected.

### 3.2 Chemogenetic reactivation of LEC learning‐tagged neurons restores OPC memory retrieval in 2‐month‐old APP mice

Based on the lack of behavioral and molecular differences during the encoding of OPC associations, it could be hypothesized that the memory deficits in young APP mice might depend on a retrieval rather than a storage impairment.

**FIGURE 2:**
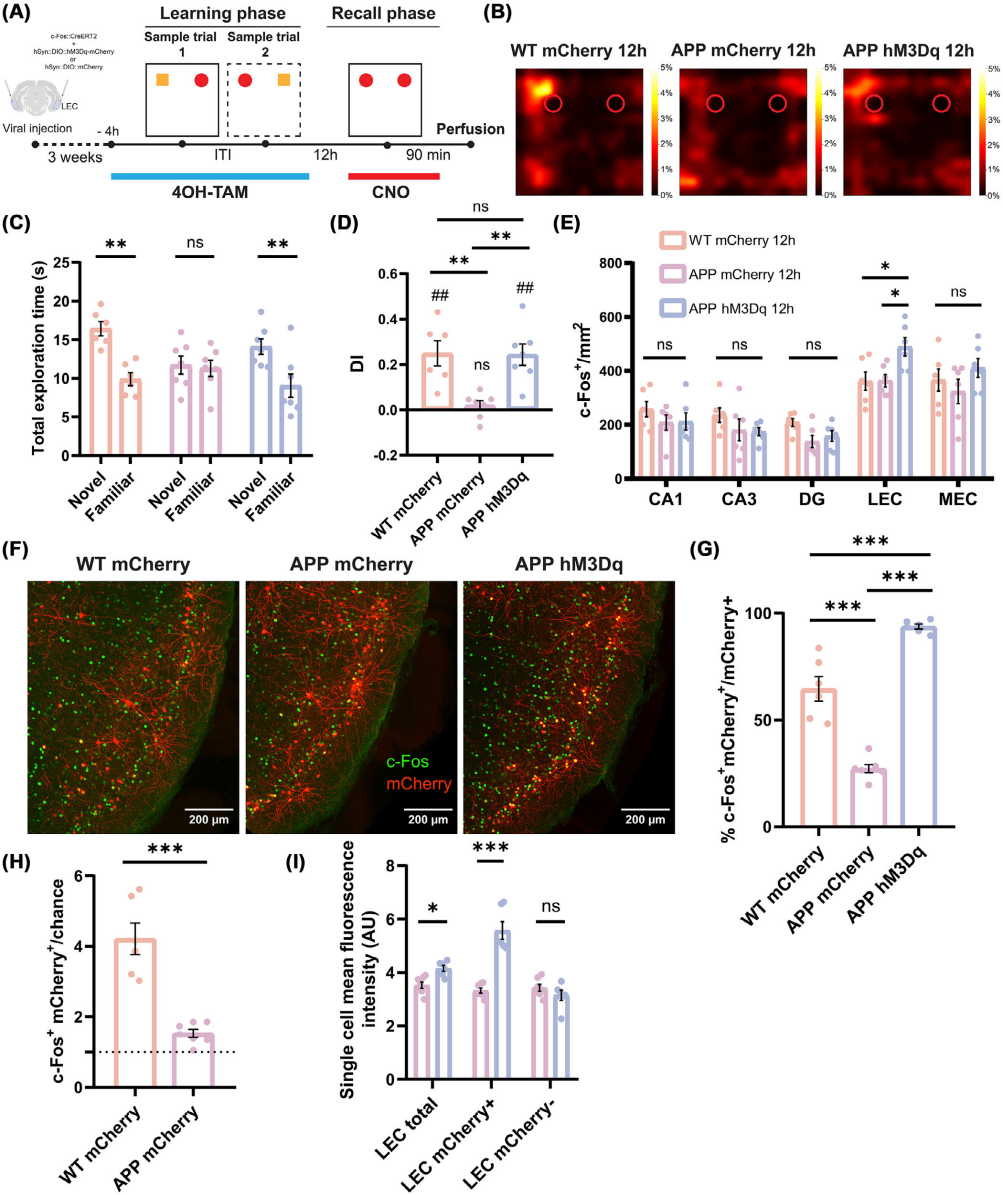
Chemogenetic reactivation of LEC learning‐tagged neurons restores object‐place‐context memory in 2‐month‐old APP mice. (A) Schematic representation of object‐place‐context recognition task: 4‐hydroxytamoxifen treatment was administered 4 h prior to the learning phase, while CNO injection was given 30 min before the recall phase. Animals were perfused 90 min after the recall phase. (B) Examples of heatmaps showing the exploratory activity of mice (nose position) during the recall phase for the three groups. WT control and APP hM3Dq mice exhibited a preference for the novel object, while APP mCherry mice explored both objects equally. The color bar represents the percentage of total time spent in each position. (C) Analysis of total exploration time revealed a significant exploration of the novel object in both the WT control and APP hM3Dq groups. In contrast, the APP mCherry control group failed to recognize the novel object during the recall phase (two‐way ANOVA with Sidak's multiple comparisons test: WT mCherry Novel 16.43 ± 0.93 *n =* 6 vs WT mCherry Familiar 9.9 ± 0.843 *n =* 6, **p = 0.0013; APP mCherry Novel 11.71 ± 1.161 *n =* 7 vs APP mCherry Familiar 11.29 ± 1.05 *n =* 7, ns *p =* 0.99; APP hM3Dq Novel 14.1 ± 0.992 *n =* 7 vs APP hM3Dq Familiar 9.053 ± 1.507 *n =* 7, **p = 0.0078). (D) The discrimination index (DI) analysis showed that the WT control and APP hM3Dq groups exhibited significant exploration of the novel object above chance levels, whereas the APP mCherry control group showed no preference (WT mCherry 0.248 ± 0.055, *n =* 6, **p = 0.0065, df = 5, *t =* 4.476; APP mCherry 0.018 ± 0.02, *n =* 7, ns *p =* 0.3983, df = 6, *t =* 0.9091; APP hM3Dq 0.242 ± 0.046, *n =* 7, **p = 0.002, df = 6, *t =* 5.183; one‐sample *t*‐test). Additionally, the DI was significantly higher in the WT control and APP hM3Dq groups compared to the APP mCherry control group (one‐way ANOVA with Sidak's multiple comparisons test: WT mCherry vs APP mCherry, **p = 0.0045; WT mCherry vs APP hM3Dq, ns *p =* 0.9996; APP mCherry vs APP hM3Dq, **p = 0.004). (E) A significant increase in c‐Fos^+^ cell density was detected in the LEC for the APP hM3Dq group compared to the WT and APP mCherry group (two‐way ANOVA with Sidak's multiple comparisons test: WT mCherry CA1 258 ± 28.55 *n =* 6 vs APP mCherry CA1 208.2 ± 27.84 *n =* 6, ns *p =* 0.4887; WT mCherry CA1 vs APP hM3Dq CA1 212.3 ± 32.15 *n =* 6, ns *p =* 0.5458; APP mCherry CA1 vs APP hM3Dq CA1, ns *p =* 0.9952; WT mCherry CA3 235.5 ± 26.88 *n =* 6 vs APP mCherry CA3 180.6 ± 40.35 *n =* 6, ns *p =* 0.4193; WT mCherry CA3 vs APP hM3Dq CA3 174.2 ± 14.99 *n =* 6, ns *p =* 0.3400; APP mCherry CA3 vs APP hM3Dq CA3, ns *p =* 0.9882; WT mCherry DG 208.5 ± 14.90 *n =* 6 vs APP mCherry DG 137.9 ± 22.58 *n =* 6, ns *p =* 0.2402; WT mCherry DG vs APP hM3Dq DG 158.5 ± 19.88 *n =* 6, ns *p =* 0.4853; APP mCherry DG vs APP hM3Dq DG, ns *p =* 0.8828; WT mCherry LEC 361.9 ± 33.59 *n =* 6 vs APP mCherry LEC 363.3 ± 23.23 *n =* 6, ns *p =* 0.9994; WT mCherry LEC vs APP hM3Dq LEC 489 ± 34.31 *n =* 6, *p = 0.0124; APP mCherry LEC vs APP hM3Dq LEC, *p = 0.0136; WT mCherry MEC 365.6 ± 40.89 *n =* 6 vs APP mCherry MEC 323.4 ± 45.13 *n =* 6, ns *p =* 0.5960; WT mCherry MEC vs APP hM3Dq MEC 410.3 ± 35.26 *n =* 6, ns *p =* 0.5603; APP mCherry MEC vs APP hM3Dq MEC, ns *p =* 0.1185). (F) Representative images of mCherry (red) and c‐Fos (green) expressing neurons in the LEC of the three groups. Scale bars are shown in the figures. (G) Co‐localization analysis of mCherry and c‐Fos signals revealed a significant reduction in the APP mCherry group compared to the WT mCherry control. In contrast, a significant increase was observed in the APP hM3Dq group, associated with the chemogenetic activation of learning‐tagged LEC neurons (one‐way ANOVA with Sidak's multiple comparisons test: WT mCherry 64.57 ± 5.748 *n =* 6 vs APP mCherry 27.17 ± 1.886 *n =* 7, ***p < 0.001; WT mCherry 64.57 ± 5.748 *n =* 6 vs APP hM3Dq 93.76 ± 1.151 *n =* 7, ***p < 0.001; APP mCherry 27.17 ± 1.886 *n =* 7 vs APP hM3Dq 93.76 ± 1.151 *n =* 7, ***p < 0.001). (H) The reactivation of the LEC learning‐tagged neurons was measured as the percentage of c‐Fos^+^ mCherry^+^ overlap over chance levels. A significant reduction of the reactivation level was observed in the APP mCherry group compared to the WT mCherry group (WT mCherry 4.216 ± 0.447 *n =* 6 vs APP mCherry 1.532 ± 0.112 *n =* 7, ***p < 0.001, df = 11, *t =* 6.262, two‐tailed unpaired *t* test). (I) A significant increase in the mean fluorescence intensity of individual cells was found in the total population of c‐Fos^+^ LEC neurons of the APP hM3Dq group compared to the APP mCherry control. This difference depends on a strong increase in the mean fluorescence of the mCherry^+^ population but not of the mCherry^−^ (two‐way ANOVA with Sidak's multiple comparisons test: APP mCherry LEC total 3.522 ± 0.1214 *n =* 7 vs APP hM3Dq LEC total 4.151 ± 0.11 *n =* 7, *p = 0.0454; APP mCherry LEC mCherry^+^ 3.324 ± 0.095 *n =* 7 vs APP hM3Dq LEC mCherry^+^ 5.572 ± 0.33 *n =* 7, ***p < 0.001; APP mCherry LEC mCherry^−^ 3.424 ± 0.13 *n =* 7 vs APP hM3Dq LEC mCherry^−^ 3.147 ± 0.191 *n =* 7, ns *p =* 0.6091). Data are presented as mean ± SEM. APP, amyloid precursor protein; CNO, clozapine N‐oxide hydrochloride; df, degrees of freedom; LEC, lateral entorhinal cortex; WT, wild type.

To investigate this issue, we applied a double AAV system based on TRAP technology to drive the expression of the excitatory chemogenetic receptor hM3Dq in the subpopulation of LEC neurons activated during the learning phase of the OPCRT.^21^ To achieve this, mice were injected with 4OH‐TAM 4 h before the learning phase to express the designer receptors exclusively activated by designer drugs (DREADDs) in the LEC neurons expressing c‐Fos during the sample trials. Then, by administering clozapine N‐oxide (CNO) 30 min before the recall phase, it was possible to selectively manipulate the activity of these neuronal ensembles to determine whether memories could be retrieved 12 h after the learning phase under chemogenetic reactivation (Figure 2A and Figure S2A).

At the behavioral level, 2‐month‐old APP control mice injected with the AAV encoding only for the mCherry reporter failed to retrieve the episodic‐like memory trace 12 h after the learning phase. However, chemogenetic activation of LEC learning‐tagged neurons in APP hM3Dq‐expressing mice rescued memory recall at 12 h. The behavioral performance was significantly different from that of the APP control mice but not different from that of the WT mCherry control (Figure 2B–D). To exclude the possibility that the observed behavioral effect was caused by differences in exploratory activity, the total distance traveled during the test trial and the number of interactions were measured in the three groups. No significant differences were found, indicating that chemogenetic manipulation did not alter motor activity or exploratory behavior (Figure S2B,C). To examine the activation state of the medial temporal lobe system following the retrieval of episodic‐like memory, the density of c‐Fos^+^ neurons was evaluated 90 min after the recall phase of the behavioral task. No significant differences between the groups were observed in the hippocampal regions, including the DG, CA1, and CA3, or in the MEC. However, in the LEC, chemogenetic activation resulted in a significant increase in the density of cFos^+^ neurons, indicating that the activation of this specific neuronal population was successfully achieved and may be associated with the observed behavioral effects (Figure 2E).

Furthermore, immunofluorescence analysis of the subpopulations of LEC learning‐tagged neurons revealed that the overlap between mCherry and c‐Fos signals was significantly lower in the APP mCherry group compared to the WT mCherry group, indicating a specific impairment of a subpopulation of neurons with respect to the whole LEC. In the group that received chemogenetic reactivation, the overlap was significantly higher and tended to be present in nearly all the tagged cells, confirming the efficacy of the genetic system and chemogenetic stimulation (Figure 2F,G). Furthermore, we did not observe any differences in the number of tagged neurons across the three groups, indicating that memory allocation during encoding is not impaired in APP mice (Figure S2D). Measuring the reactivation of LEC learning‐tagged neurons as the percentage of c‐Fos^+^ and mCherry^+^ overlap over chance levels, it was possible to observe that the successful natural cue‐induced recall in the WT control was associated with a significative higher reactivation rate compared to the APP mCherry control (Figure 2H). It has already been demonstrated that in WT mice, the overlap between mCherry and c‐Fos significantly correlated with the DI.^21^ In contrast, it was not possible to detect any significant correlation in the APP mCherry group (Figure S2E).

Similarly to the analysis of cFos^+^ density, the mean fluorescence intensity per cell did not differ in the hippocampus and MEC (Figure S2F). In the LEC, a significant increase in mean fluorescence intensity was observed in the APP hM3Dq group compared to the APP mCherry control. This effect was driven by a significant increase in the mean fluorescence of the sub‐population of mCherry^+^ neurons activated through chemogenetic manipulation. The remaining mCherry^−^ population in the LEC was unaffected by chemogenetic activation, suggesting that this effect is specific to the manipulated neuronal ensemble (Figure 2I). Consistently, whole‐cell patch‐clamp recordings further confirmed that LEC neurons of the hM3Dq group exhibited a significant response to CNO administration, characterized by a depolarization of the resting membrane potential and a marked increase in excitability (Figure S3A–C). Specifically, CNO‐treated neurons displayed an increase in both the amplitude and frequency of spontaneous synaptic activity (Figure S3D–G), along with a step‐dependent increase in action potential firing in response to depolarizing current injections (Figure S3H,I).

Interestingly, chemogenetic reactivation of learning‐tagged ensembles in the DG at both 2 m.o. and 6 m.o. failed to rescue behavioral performance, suggesting that the reactivation of LEC learning‐tagged ensembles is necessary for the recall of associative object‐place‐context memory (Figure S4A–D).

These findings suggest that young APP mice exhibit a specific deficit in the reactivation of the subpopulation of LEC neurons recruited during the learning phase of the OPCRT. Chemogenetic manipulation successfully rescued memory performance, indicating that OPC memory is encoded in APP mice, but not accessible through natural cues alone.

### 3.3 Chemogenetic reactivation of learning‐tagged neurons promotes a shift in dendritic spine morphology

**FIGURE 3:**
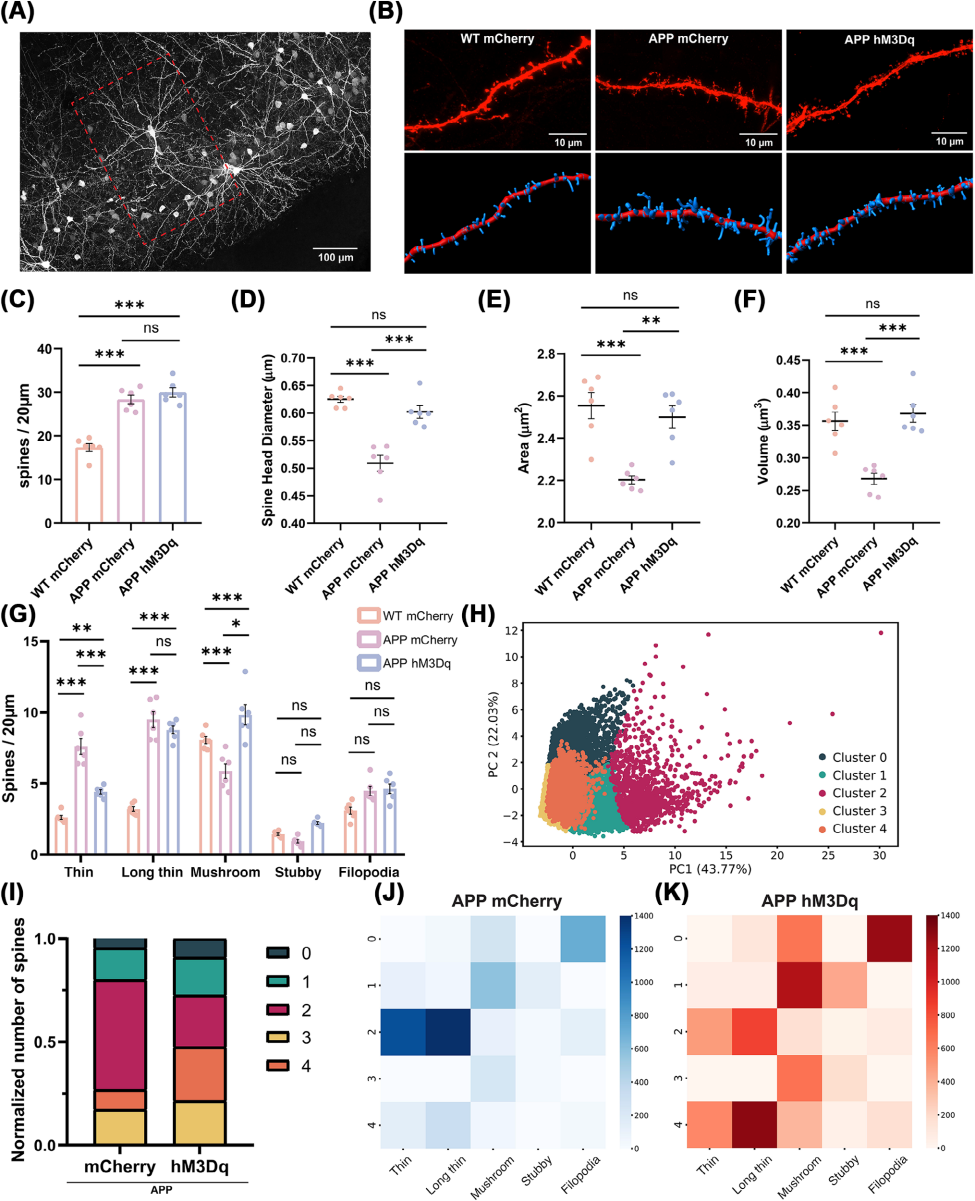
Chemogenetic reactivation of learning‐tagged neurons promotes a shift in dendritic spine morphology. (A) Representative image showing an EC pyramidal neuron in layer II/III from the population of mCherry^+^ learning‐tagged neurons, captured at 40× magnification. (B) Representative images of selected dendrites at 63× magnification, with corresponding 3D reconstructions shown below. (C) Spine density was significantly lower in WT mCherry compared to APP groups, with no difference between APP mCherry and hM3Dq (one‐way ANOVA with Sidak's multiple comparisons test: WT mCherry 17.39 ± 0.899 *n =* 6 vs APP mCherry 28.38 ± 1.019 *n =* 6, ***p < 0.001; WT mCherry vs APP hM3Dq 29.99 ± 1.066, ***p < 0.001; APP mCherry vs APP hM3Dq, ns *p =* 0.61). (D) A significant reduction in spine head diameter was detected in the APP mCherry group compared to both WT mCherry and APP hM3Dq (one‐way ANOVA with Sidak's multiple comparisons test: WT mCherry 0.624 ± 0.005 *n =* 6 vs APP mCherry 0.509 ± 0.015 *n =* 6, ***p < 0.001; WT mCherry vs APP hM3Dq 0.602 ± 0.011, ns *p =* 0.4554; APP mCherry vs APP hM3Dq, ***p < 0.001). (E) A significant reduction in spine area was detected in the APP mCherry group compared to both WT mCherry and APP hM3Dq (one‐way ANOVA with Sidak's multiple comparisons test: WT mCherry 2.554 ± 0.061 *n =* 6 vs APP mCherry 2.203 ± 0.019 *n =* 6, ***p < 0.001; WT mCherry vs APP hM3Dq 2.501 ± 0.053, ns *p =* 0.8254; APP mCherry vs APP hM3Dq, ***p < 0.001). (F) A significant reduction in spine volume was detected in the APP mCherry group compared to both WT mCherry and APP hM3Dq (one‐way ANOVA with Sidak's multiple comparisons test: WT mCherry 0.356 ± 0.014 *n =* 6 vs APP mCherry 0.267 ± 0.008 *n =* 6, ***p < 0.001; WT mCherry vs APP hM3Dq 0.3681 ± 0.013, ns *p =* 0.8814; APP mCherry vs APP hM3Dq, ***p < 0.001). (G) Using a five‐category classification, it was possible to observe a significant decrease in thin spines and a concomitant increase in mushroom spines in hM3Dq mice. Data are shown as the number of spines per 20 µm (two‐way ANOVA with Sidak's multiple comparisons test: APP mCherry thin 7.592 ± 0.549 *n =* 7 vs APP hM3Dq thin 4.407 ± 0.151 *n =* 7, ***p < 0.001; APP mCherry long thin 9.497 ± 0.552 *n =* 7 vs APP hM3Dq long thin 8.773 ± 0.277 *n =* 7, ns *p =* 0.7134; APP mCherry mushroom 5.873 ± 0.508 *n =* 7 vs APP hM3Dq mushroom 9.829 ± 0.707 *n =* 7, ***p < 0.001; APP mCherry stubby 0.936 ± 0.126 *n =* 7 vs APP hM3Dq stubby 2.224 ± 0.089 *n =* 7, ns *p =* 0.1507; APP mCherry filopodia 4.478 ± 0.318 *n =* 7 vs APP hM3Dq filopodia 4.625 ± 0.337 *n =* 7, ns *p =* 0.9997). (H) PCA‐based clustering of the dataset using *k*‐means. The scatter plot represents the first two principal components (PC1 and PC2) of the scaled data, with each point color‐coded according to its cluster assignment. The clusters were determined using *k*‐means (*k* = 5), and distinct colors are used to represent each cluster. The percentage of variance explained by each principal component is indicated on the axes. On the right, cluster proportions for mCherry and hM3Dq groups. (I) Stacked bar plot displaying the proportion of data points in each cluster following *k*‐means clustering. For both mCherry and hM3Dq groups, the proportions reflect the relative distribution of data points across clusters, normalized to the total number of spines in the dataset. (J and K) Heatmaps showing the absolute abundance of spines across morphological categories (thin, long thin, mushroom, stubby, and filopodia) coupled with clusters identified through the unsupervised approach (PCA and *k*‐means). (J) APP mCherry group. (K) APP hM3Dq group. Color intensity indicates the absolute number of spines within each morphological category for a given cluster. The clusters are shown on the *y*‐axis, and the spine classifications are shown on the *x*‐axis. Spine morphological features are presented in µm. Data are presented as mean ± SEM. APP, amyloid precursor protein; EC, entorhinal cortex; PCA, principal component analysis; WT, wild type.

It has been demonstrated that the behavioral deficits in young APP mice are associated with the aberrant production of thin dendritic spines in layer II neurons of the LEC.^17^ To investigate differences between WT and APP mice, as well as the effect of chemogenetic stimulation on dendritic spines, we examined the morphology of apical dendritic spines on learning‐tagged pyramidal neurons in layer II/III. Changes in their structure and density were analyzed following chemogenetic reactivation through 3D reconstruction of the selected dendrites (Figure 3A,B). Interestingly, while this analysis revealed no significant changes in spine length or spine neck diameter (Figure S5A,B), we observed a significant increase in spine density in the APP group compared to WT controls (Figure 3C), a result consistent with previous reports in this model.^1^ Compared to the WT group, APP mCherry controls showed a significant reduction in spine head diameter, spine area, and spine volume. Chemogenetic activation of the learning‐tagged population in APP mice restored these parameters to WT levels (Figure 3D–F). These findings suggest a structural remodeling of the dendritic spines on LEC learning‐tagged pyramidal neurons, associated with chemogenetic stimulation. To further explore the morphological alterations, we classified the dendritic spines according to the parameters shown in Figure S5C. This classification enabled a more precise categorization of the spines, offering a clearer insight into the structural diversity and the potential effects of chemogenetic manipulation on spine morphology. Comparison of WT controls with the APP mCherry group revealed a significantly higher proportion of thin and long thin spines in APP neurons, whereas mushroom spines were more prevalent in WT neurons, suggesting that APP mice exhibit an enrichment of immature and potentially dysfunctional spines. Following chemogenetic stimulation, we observed a significant reduction in thin spines and a marked increase in mushroom‐shaped spines (Figure 3G). This shift in spine morphology suggests a remodeling process, where the smaller spines are replaced by the more stable and functionally mature mushroom spines, which may provide increased stability through their larger size and potentially higher AMPA receptor content. To further investigate the potential morphological changes following chemogenetic activation in the APP groups, we employed an unsupervised method to classify dendritic spines based on their morphological features, allowing for an objective and comprehensive analysis of spine characteristics. As a first step in the analysis, we applied PCA to reduce the dimensionality of 16 features extracted from the 3D reconstruction of dendritic spines. We then performed *k*‐means clustering to identify distinct groups based on the morphological characteristics of the spines (Figure 3H). The analysis of the first two components revealed that a clear separation of the data was not possible, likely due to the continuous variation in the morphological properties of the dendritic spines. However, we observed a shift in the relative abundance of spines across the five clusters between the APP mCherry and APP hM3Dq groups. Notably, this variation was especially evident in Clusters 2 and 4. In APP hM3Dq, Cluster 2 showed a marked reduction in spine abundance, while Cluster 4 exhibited a substantial increase. These changes suggest that chemogenetic stimulation may selectively influence the distribution of dendritic spine subtypes, potentially reflecting a remodeling of dendritic architecture in response to the stimulation (Figure 3I).

To better understand the relationship between the clusters identified through the unsupervised method and the spine types defined through the supervised classification, we plotted the clusters alongside the original spine classification. The analysis revealed that the clusters identified through the unsupervised method correspond to distinct morphological spine types. Specifically, Cluster 0 predominantly represents long spines, such as filopodia, while Cluster 2 is enriched with thin and long thin spines. Clusters 0, 1, and 3 collectively represent mushroom spines, likely reflecting the observed increase in head diameter of these spines. In the APP hM3Dq group, we observed a reduction in spines within Cluster 2, consistent with a decrease in thin spines and a concurrent increase in spines across clusters representing mushroom spines. Interestingly, the APP hM3Dq group also exhibited an increase in spines within Cluster 4, which appears to represent a transitional state between long thin and mushroom spines. This transitional state is not fully captured by the supervised classification, highlighting the added value of the unsupervised approach in identifying intermediate morphological changes that may play a role in dendritic spine remodeling and synaptic plasticity (Figure 3J,K). Taken together, our findings provide evidence for a structural remodeling of dendritic spines in response to chemogenetic stimulation. The combination of supervised and unsupervised analyses revealed a shift in the distribution of spine subtypes, with a reduction in thin spines and an increase in mushroom spines, alongside the emergence of a transitional spine population. These findings suggest that the memory rescue induced by chemogenetic stimulation in early stage APP mice is associated with a facilitation of the transition toward a more mature and stable spine state in LEC neurons, as evidenced by structural enlargement.

### 3.4 Impaired hippocampal c‐Fos activation following behavioral stimulation in 6‐month‐old APP mice

**FIGURE 4:**
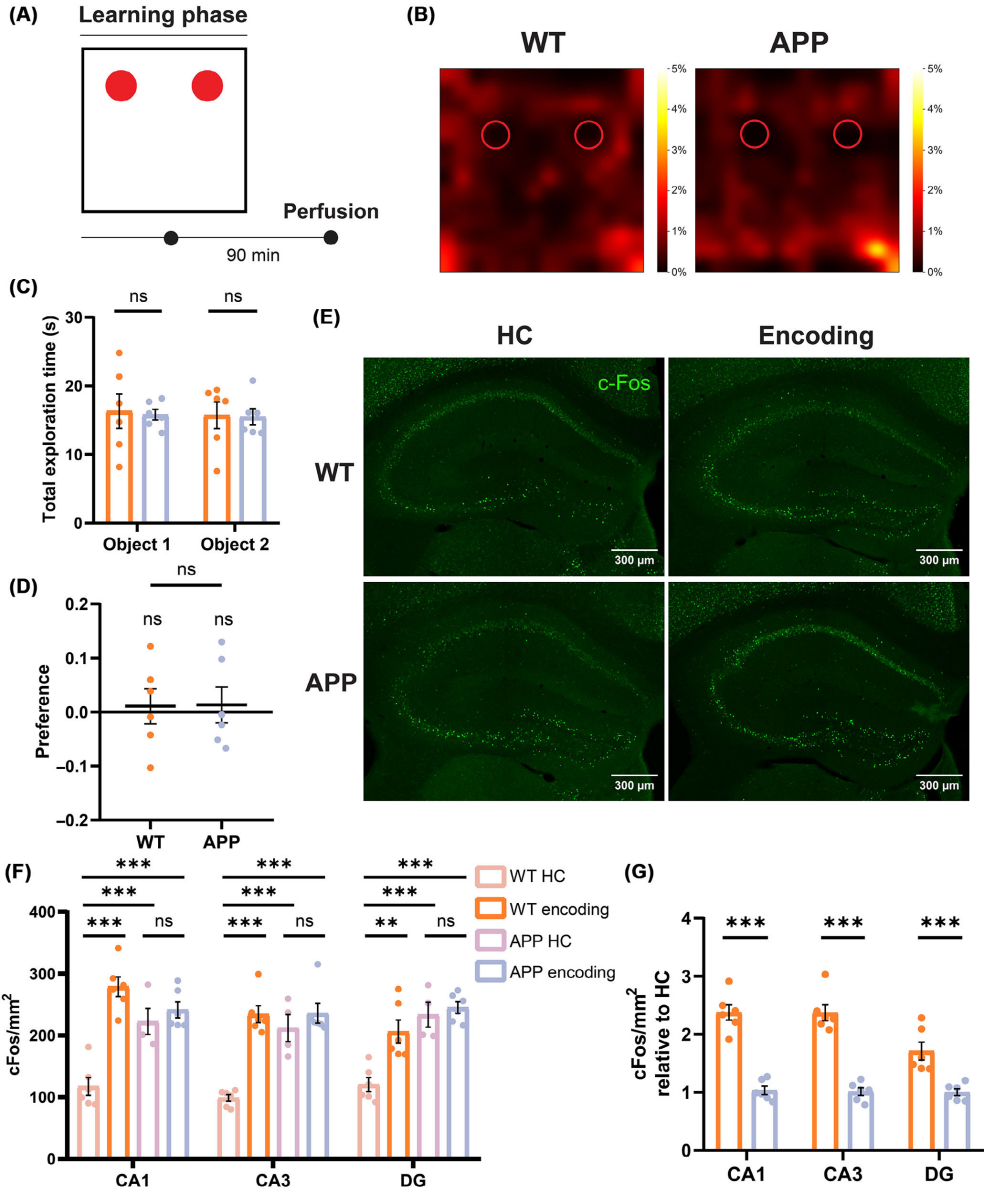
Impaired hippocampal c‐Fos activation following behavioral stimulation in 6‐month‐old APP mice. (A) Schematic representation of the learning phase of the ORT. Animals were perfused 90 min after the end of the task. (B) Examples of heatmaps showing the exploratory activity of mice (nose position) during the task. 6‐month‐old WT control and APP mice exhibited no preference for either object. (C) Analysis of total exploration time revealed that mice spent a comparable amount of time interacting with both objects (two‐way ANOVA with Sidak's multiple comparisons test: WT encoding Object 1 16.32 ± 2.524 *n =* 6 vs APP encoding Object 1 15.8 ± 0.778 *n =* 7, ns *p =* 0.9724; WT encoding Object 2 15.71 ± 1.925 *n =* 6 vs APP encoding Object 2 15.49 ± 1.189 *n =* 7, ns *p =* 0.9951). (D) During the learning phase, mice showed no preference for either object, with exploration not differing from chance (WT encoding 0.01 ± 0.032, *n =* 6, ns *p =* 0.7511, df = 5, *t =* 0.3352; APP encoding 0.013 ± 0.033, *n =* 6, ns *p =* 0.7049, df = 5, *t =* 0.4012; one‐sample *t* test) or between groups (WT encoding vs APP encoding, ns *p =* 0.9594, df = 10, *t =* 0.0521, two‐tailed unpaired *t* test). (E) Representative images showing c‐Fos expression (green) in the hippocampus of 6‐month‐old WT and APP mice in the HC control condition or following behavioral stimulation during the learning phase of the ORT. Scale bars are included in the figures. (F) Analysis of c‐Fos+ cell density revealed a specific alteration in c‐Fos expression in 6‐month‐old APP mice. While WT mice exhibited a significant increase in c‐Fos density across the hippocampus following behavioral stimulation, APP mice displayed high basal HC levels of c‐Fos protein, which remained unchanged after the task. Moreover, c‐Fos levels in APP mice, both in the HC and after encoding, did not differ from those observed in the WT encoding group (two‐way ANOVA with Sidak's multiple comparisons test: WT HC CA1 117.2 ± 14.43 *n =* 6 vs WT encoding CA1 278.7 ± 15.81 *n =* 6, ***p < 0.001; WT HC CA1 vs APP HC CA1 206.1 ± 18.65 *n =* 4, ***p < 0.001; WT HC CA1 vs APP encoding CA1 241.2 ± 12.90 *n =* 6, ***p < 0.001; WT encoding CA1 vs APP encoding CA1, ns *p =* 0.2451; APP HC CA1 vs APP encoding CA1, ns p > 0.9999; WT HC CA3 98.75 ± 5.371 *n =* 6 vs WT encoding CA3 234.2 ± 13.71 *n =* 6, ***p < 0.001; WT HC CA3 vs APP HC CA3 211.8 ± 21.81 *n =* 4, ***p < 0.001; WT HC CA3 vs APP encoding CA3 235.8 ± 15.99 *n =* 6, ***p < 0.001; WT encoding CA3 vs APP encoding CA3, ns *p* > 0.9999; APP HC CA3 vs APP encoding CA3, ns *p =* 0.9979; WT HC DG 92.17 ± 11.29 *n =* 6 vs WT encoding DG 206.1 ± 18.65 *n =* 6, **p = 0.0015; WT HC DG vs APP HC DG 233.4 ± 20.02 *n =* 4, ***p < 0.001; WT HC DG vs APP encoding DG 245 ± 9.417 *n =* 6, ***p < 0.001; WT encoding DG vs APP encoding DG, ns *p =* 0.6615; APP HC DG vs APP encoding DG, ns p > 0.9999). (G) Analysis of hippocampal c‐Fos^+^ density relative to home cage control (HC) revealed a lack of c‐Fos protein increase after the encoding phase in APP mice compared to WT controls. Each encoding group was normalized to the mean c‐Fos^+^ density of its respective HC control of the same genotype (two‐way ANOVA with Sidak's multiple comparisons test: WT encoding CA1 2.377 ± 0.134 *n =* 6 vs APP encoding CA1 1.033 ± 0.071 *n =* 6, ***p < 0.001; WT encoding CA3 2.372 ± 0.138 *n =* 6 vs APP encoding CA3 1.014 ± 0.065 *n =* 6, ***p < 0.001; WT encoding DG 1.711 ± 0.154 *n =* 6 vs APP encoding DG 1.004 ± 0.058 *n =* 6, ***p < 0.001). APP, amyloid precursor protein; df, degrees of freedom; DG, dentate gyrus; HC, home cage; ORT, object recognition test; WT, wild type.

Previous studies have demonstrated that at a later stage of neurodegeneration (i.e., 6 months old), APP mice displayed a significant deficit in remembering the familiar object in the less cognitively demanding novel ORT, which is indicative of the progression of amyloid pathology from the EC to the hippocampus.^17^ To determine whether the deficit in object recognition memory recall is linked to changes during object recognition encoding, we evaluated behavioral performance and hippocampal neuronal activation during the learning phase of the ORT (Figure 4A). Despite impaired memory performance during the recall phase, 6‐month‐old APP mice did not exhibit any significant alteration in behavioral performance during the learning phase. Both 6‐month‐old APP and WT mice showed no preference for the objects in either position (Figure 4B‐D). Distance analysis revealed no significant differences between the groups at this age (Figure S6A). Similarly, APP mice showed no differences in the number of object interactions (Figure S6B). These findings suggest that during the learning phase of the novel ORT, no detectable changes in behavioral performance were observed in 6‐month‐old APP mice compared to WT. To investigate whether the encoding of a non‐associative memory is associated with a similar pattern of activation in hippocampal subareas, we analyzed the density and fluorescence relative intensity of c‐Fos^+^ neurons 90 min after the encoding phase of the novel ORT. To assess the c‐Fos expression response to behavioral stimulation in both groups, we also evaluated baseline c‐Fos protein expression in the hippocampus of home cage controls (Figure 4E,F). Notably, while 6‐month‐old WT mice exhibited a significant activation pattern in CA1, CA3, and DG compared to their home cage controls, APP mice showed high activity levels in the home cage but lacked activation following the behavioral task. The basal level of c‐Fos^+^ expression in APP mice was significantly higher than that of WT mice and comparable to the activation observed after the encoding phase in WT mice. This was particularly evident when examining the density of c‐Fos^+^ cells relative to home cage conditions, which revealed a lack of response to behavioral stimulation (Figure 4G). Interestingly, no significant differences were observed in the mean single‐cell fluorescence intensity between groups (Figure S6C). These findings collectively indicate that 6‐month‐old APP mice exhibit an elevated basal level of c‐Fos expression in the hippocampus. Notably, this expression level remained unchanged following the encoding of the novel ORT, suggesting an impaired ability to modulate hippocampal activation in response to behavioral stimuli.

### 3.5 Chemogenetic reactivation of DG learning‐tagged neurons rescues novel object recognition in 6‐month‐old APP mice

**FIGURE 5:**
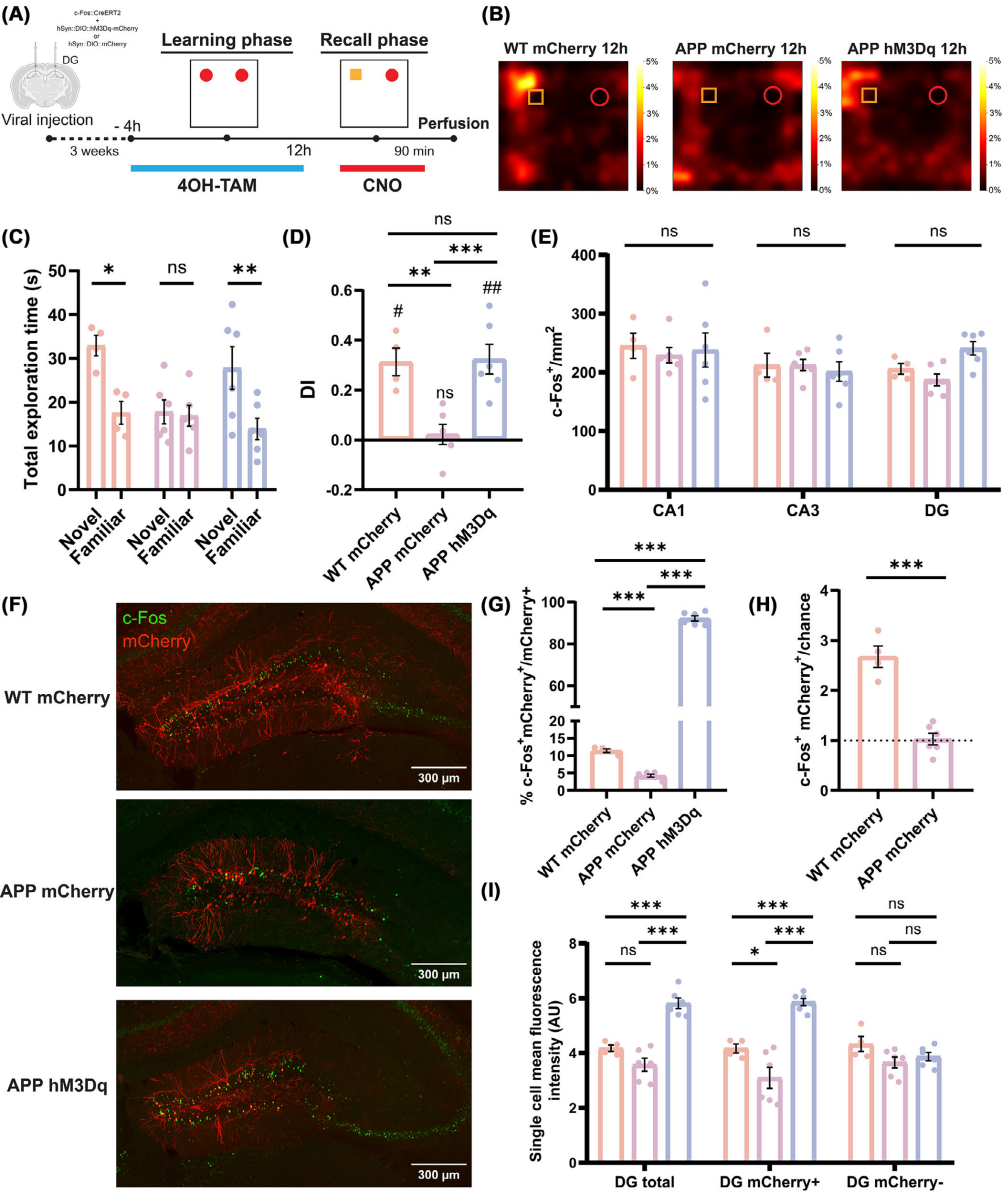
Chemogenetic reactivation of dentate gyrus learning‐tagged neurons rescues novel object recognition in 6‐month‐old APP mice. (A) Schematic representation of the ORT: 4‐hydroxytamoxifen treatment was administered 4 h prior to the learning phase, while clozapine N‐oxide hydrochloride injection was given 30 min before the recall phase. Animals were perfused 90 min after the recall phase. (B) Examples of heatmaps showing the exploratory activity of mice (nose position) during the recall phase for the three groups. WT control and APP hM3Dq mice showed a preference for the novel object, whereas APP mCherry mice explored both objects equally. The color bar represents the percentage of total time spent in each position. (C) Total exploration time analysis showed significant exploration of the novel object in both the WT control and APP hM3Dq groups. In contrast, the APP mCherry control group did not recognize the novel object during the recall phase (two‐way ANOVA with Sidak's multiple comparisons test: WT mCherry Novel 32.92 ± 2.321 *n =* 6 vs WT mCherry Familiar 17.57 ± 2.609 *n =* 4, *p = 0.0.0210; APP mCherry Novel 17.78 ± 2.736 *n =* 6 vs APP mCherry Familiar 16.90 ± 2.376 *n =* 6, ns *p =* 0.9958; APP hM3Dq Novel 27.81 ± 4.875 *n =* 6 vs APP hM3Dq Familiar 13.90 ± 2.442 *n =* 6, **p = 0.0096). (D) The DI analysis revealed that the WT control and APP hM3Dq groups significantly explored the novel object above chance levels, while the APP mCherry control group showed no preference (WT mCherry 0.3127 ± 0.055, *n =* 4, **p = 0.0110, df = 3, *t =* 5.643; APP mCherry −0.022 ± 0.040, *n =* 6, ns *p =* 0.6051, df = 5, *t =* 0.5514; APP hM3Dq 0.3238 ± 0.059, *n =* 6, **p = 0.0029, df = 5, *t =* 5.434; one‐sample *t* test). Furthermore, the DI was significantly higher in both the WT control and APP hM3Dq groups compared to the APP mCherry control group (one‐way ANOVA with Sidak's multiple comparisons test: WT mCherry vs APP mCherry, **p = 0.008; WT mCherry vs APP hM3Dq, ns *p =* 0.9987; APP mCherry vs APP hM3Dq, **p = 0.0026). (E) No significant differences in c‐Fos cell density were detected in the regions of interest across the three groups. A trend toward higher values was observed in the DG of APP hM3Dq, which may be associated with the chemogenetic activation of subpopulations of learning‐tagged DG neurons (two‐way ANOVA with Sidak's multiple comparisons test: WT mCherry CA1 245.1 ±  21.51 *n =* 4 vs APP mCherry CA1 229.1 ± 13.10 *n =* 6, ns *p =* 0.8050; WT mCherry CA1 vs APP hM3Dq CA1 238 ± 29.08 *n =* 6, ns *p =* 0.9576; APP mCherry CA1 vs APP hM3Dq CA1, ns *p =* 0.9197; WT mCherry CA3 212.1 ± 20.34 *n =* 4 vs APP mCherry CA3 212.4 ± 9.49 *n =* 6, ns *p* > 0.9999; WT mCherry CA3 vs APP hM3Dq CA3 201.5 ± 16.68 *n =* 6, ns *p =* 0.9089; APP mCherry CA3 vs APP hM3Dq CA3, ns *p =* 0.8814; WT mCherry DG 205.8 ± 9.02 *n =* 4 vs APP mCherry DG 187.2 ± 10.08 *n =* 6, ns *p =* 0.7450; WT mCherry DG vs APP hM3Dq DG 240.9 ± 11.65 *n =* 6, ns *p =* 0.3591; APP mCherry DG vs APP hM3Dq DG, ns *p =* 0.0583). (F) Representative images of mCherry (red) and c‐Fos (green) expressing neurons in the DG of the WT mCherry, APP mCherry, and APP hM3Dq groups. Scale bars are shown in the figures. (G) Co‐localization analysis of mCherry and c‐Fos signals showed a significant reduction in the APP mCherry group compared to the WT mCherry control (WT mCherry 11.44 ± 0.0548 *n =* 4 vs APP mCherry 4.261 ± 0.4206, ***p < 0.001, df = 8, *t =* 10.53, two‐tailed unpaired *t* test). (H) The reactivation of LEC learning‐tagged neurons was assessed as the percentage of c‐Fos^+^ mCherry^+^ overlap relative to chance levels. A significant reduction in reactivation was observed in the APP mCherry group compared to the WT mCherry group (WT mCherry 2.677 ± 0.2147 *n =* 4 vs APP mCherry 1.028 ± 0.1168, ***p < 0.001, df = 8, *t =* 7.365, two‐tailed unpaired *t* test). (I) A significant increase in the mean relative fluorescence intensity was observed in the total population of c‐Fos^+^ DG neurons in the APP hM3Dq group compared to the APP mCherry control. This difference was driven by a marked increase in the mean fluorescence of the mCherry^+^ population, with no change in the mCherry^−^ population (two‐way ANOVA with Sidak's multiple comparisons test: WT mCherry DG total 4.177 ± 0.118 *n =* 4 vs APP mCherry DG total 3.577 ± 0.2366 *n =* 6, ns *p =* 0.5672; WT mCherry DG total vs APP hM3Dq DG total 5.814 ± 0.1939 *n =* 6, ***p < 0.001; APP mCherry DG total vs APP hM3Dq DG total, ***p < 0.001; WT mCherry DG mCherry^+^ 4.170 ± 0.165 *n =* 4 vs APP mCherry DG mCherry^+^ 3.099 ± 0.384 *n =* 6, *p = 0.0307; WT mCherry DG mCherry^+^ vs APP hM3Dq DG mCherry^+^ 5.862 ± 0.133 *n =* 6, ***p < 0.001; APP mCherry DG mCherry^+^ vs APP hM3Dq DG mCherry^+^, ***p < 0.001; WT mCherry DG mCherry^−^ 4.328 ± 0.273 *n =* 4 vs APP mCherry DG mCherry^−^ 3.659 ± 0.2024, ns *p =* 0.4209 *n =* 6; WT mCherry DG mCherry^−^ vs APP hM3Dq DG mCherry^−^ 3.869 ± 0.151 *n =* 6, ns *p =* 0.8494; APP mCherry DG mCherry^−^ vs APP hM3Dq DG mCherry^−^, ns *p =* 0.9980). Data are presented as mean ± SEM. APP, amyloid precursor protein; df, degrees of freedom; DG, dentate gyrus; DI, discrimination index; ORT, object recognition test; WT, wild type.

It has been shown that with the progression of the neurodegeneration, APP mice display diffuse amyloid immunoreactivity in the DG associated with a deficit in hippocampal synaptic plasticity and a consistent impairment in the ability to correctly perform the novel object recognition test.^16^, ^17^, ^27^ To assess whether object recognition memory deficits could be rescued at this age through artificial manipulation of hippocampal activity, we employed the TRAP‐based AAV system described earlier. Memory performance was evaluated 12 h after the learning phase, following CNO administration (Figure 5A). Consistent with the hypothesis that neurodegeneration progresses to the hippocampus at this stage, 6‐month‐old APP mice (APP mCherry control group) exhibited impaired novel object recognition, failing to preferentially recognize and interact with the novel object during the recall phase. In contrast, 6‐month‐old WT mice (WT mCherry control group) performed correctly 12 h after the learning phase. Notably, reactivation of the subpopulation of DG learning‐tagged neurons restored a strong preference for the novel object, as indicated by both the total exploration time and the DI (Figure 5B‐D). The observed effect was not associated with a significant change in locomotor activity or the number of interactions with the object, suggesting that the chemogenetic manipulation did not influence anxiety levels (Figure S7A,B).

Consistent with the activation profile observed during the learning phase, the analysis of c‐Fos^+^ neuronal density after the task showed no significant differences in the hippocampus of APP mice compared to the WT control group. The chemogenetic activation of the DG was associated with a slight, but non‐significant, increase in c‐Fos^+^ cell density (*p =* 0.0583) likely associated with the artificial activation of the sparse subpopulation of tagged neurons (Figure 5E). Immunofluorescence analysis of DG learning‐tagged neurons (Figure 5F) revealed a significant reduction in the overlap between mCherry and c‐Fos signals in the APP mCherry group compared to the WT mCherry group (Figure 5G). This suggests a deficit in the reactivation of this specific neuronal subpopulation in the DG of 6‐month‐old APP mice. No differences in the number of tagged neurons were detected across groups, indicating intact memory allocation during the encoding in APP mice (Figure S7C). Additionally, measuring the reactivation above chance level revealed a significantly higher reactivation rate in the WT mCherry group compared to the APP mCherry control group (Figure 5H). Looking at the mean single‐cell fluorescence intensity, it was not possible to observe differences in CA1 and CA3 in the three groups (Figure S7D). In the DG, the APP hM3Dq group exhibited a significant increase in mean fluorescence intensity compared to both the APP mCherry and WT mCherry control groups. This effect, similar to what was observed in the LEC, was primarily driven by a substantial increase in mean fluorescence within the mCherry^+^ subpopulation of neurons activated through chemogenetic manipulation. In contrast, the mCherry^−^ population in the DG showed no such changes, indicating that the observed effect is specific to the subpopulation activated by the DREADDs (Figure 5I). These data indicate that 6‐month‐old APP mice exhibit a disruption in the reactivation mechanism of the DG neuronal subpopulation engaged during the learning phase of a non‐associative memory. Chemogenetic reactivation of DG learning‐tagged neurons in 6‐month‐old APP mice is sufficient to restore memory retrieval. This finding suggests that during progressive Aβ accumulation, the memory trace becomes inaccessible rather than lost.

## 4 DISCUSSION

AD is characterized by progressive cognitive decline, with episodic memory impairment emerging as one of the earliest symptoms.^28^ These deficits are associated with the disruptions of neural circuits involving the EC and hippocampus, regions that are critical for the integration of contextual details of an experience.^29^ Our findings provide compelling evidence that memory deficits in APP mice stem from impairments in memory retrieval rather than storage, reinforcing the hypothesis that early AD pathology disrupts the mechanisms necessary for accessing previously encoded information, consistent with findings previously reported in the DG.^18^ Following the well‐established progression of neurodegeneration in AD,^11^, ^29^ we initially focused on LEC in 2‐month‐old APP mice as a model of early neurodegeneration within the medial temporal lobe. In fact, it has already been shown that LEC synaptic function is affected early in APP mice, leading to a specific impairment in OPC recognition memory. This effect is associated with the accumulation of Aβ, which induces pro‐inflammatory factors and activates stress‐related protein kinases such as c‐Jun N‐terminal kinase (JNK) and p38 MAPK, contributing to synaptic dysfunction and memory deficits.^17^

Numerous studies suggest that the MEC plays a key role in constructing a universal allocentric map,^30^, ^31^ supported by many specialized cell types such as grid and boundary cells. The LEC exhibits limited allocentric integration but receives rich multisensory inputs and actively encodes spatial information linked to objects in the environment.^32^, ^33^ The LEC appears to represent this information in an egocentric coordinate frame, maintaining a flexible, spatially and temporally multiplexed code of information.^34^, ^35^, ^36^, ^37^ Moreover, recent findings suggest the existence of a neuronal population within the LEC that exhibits characteristics of a memory engram, particularly for OPC recognition memory.^8^, ^21^ We demonstrated that 2‐month‐old APP mice exhibit normal exploratory behavior during the learning phase of the OPCRT, which is associated with the activation of the immediate early gene *c‐fos* in the EC and hippocampus compared to home‐cage control mice. However, c‐Fos expression provides only a discrete measure of neuronal activation and does not capture potential alterations in the quality of information processing, such as changes in spike frequency or oscillatory coupling. Thus, while our results suggest that OPC association encoding might be preserved at this early disease stage, more temporally precise approaches would be required to fully assess the functional integrity of encoding mechanisms in APP mice.

The pattern of c‐Fos protein staining did not differ from that observed in age‐matched WT controls, suggesting that early Aβ accumulation may not affect the response to behavioral stimuli. However, this evidence alone is not sufficient to demonstrate that APP mice learn the association between the objects and the context. Instead, it suggests that the neural mechanisms underlying the encoding of environmental stimuli remain functional. To further explore the hypothesis that episodic memory retrieval deficits stem from impaired accessibility of the memory trace, we combined the TRAP method^20^ with a chemogenetic approach.^21^ Using a double viral system, we expressed the excitatory chemogenetic receptor hM3Dq in the subpopulation of LEC neurons activated during the learning phase of the OPCRT. This strategy allowed us to demonstrate that chemogenetic activation of these specific LEC neurons is sufficient to rescue OPC recognition memory performance in 2‐month‐old APP mice. Immunofluorescence analysis further confirmed that the memory rescue observed in APP mice was associated with a selective reactivation of the LEC neuronal ensemble engaged during learning. Specifically, we found that the overlap between mCherry and c‐Fos signals was significantly lower in APP mice compared to WT controls, indicating a specific impairment in the reactivation of the neuronal subpopulation involved in encoding OPC associations. This disruption suggests that memory deficits in APP mice are linked to a failure in the endogenous reactivation of learning‐relevant LEC neurons. Patch‐clamp recordings confirmed that in APP hM3Dq, LEC neurons respond to CNO with a depolarization of the resting potential and increased excitability. This functional validation indicates that chemogenetic activation facilitates reactivation of the tagged ensemble, likely contributing to the restoration of memory retrieval in APP mice. Consistent with this idea, the chemogenetic reactivation of DG learning‐tagged ensembles did not rescue OPC memory in either 2‐ or 6‐month‐old APP mice. This finding supports the hypothesis that the OPC association is primarily encoded within the LEC ensemble. The DG may provide critical support by integrating spatial and contextual features and relaying them to downstream hippocampal circuits, driving the emergence of a cohesive memory representation. However, in the absence of appropriate LEC reactivation, stimulation of the DG ensemble alone is not sufficient to recall the object‐place‐context association.

To further investigate the mechanisms underlying memory impairments and rescue, we examined dendritic spines, which serve as critical hubs of synaptic plasticity and neuronal function.^38^ Dendritic spines are highly dynamic structures that undergo activity‐dependent remodeling, playing a fundamental role in encoding, storing, and retrieving information.^39^, ^40^ Their morphology and density are strongly linked to synaptic strength, with structural changes reflecting modifications in synaptic connectivity.^41^ Notably, previous studies demonstrated an increased proportion of thin spines on LEC neurons in APP mice,^17^ a phenotype associated with immature or dysfunctional synapses.^42^ These structural changes may contribute to impaired synaptic stability and impaired memory retrieval. We found that chemogenetic activation of learning‐tagged neurons was associated with structural enlargement of dendritic spines and a shift toward a more mature morphology. This evidence supports the idea that targeted chemogenetic activation of LEC neurons can restore synaptic properties critical for memory retrieval, and it is consistent with several studies highlighting the importance of dendritic spines in memory processing. In agreement with this idea, Roy and colleagues^18^ demonstrated that optogenetic induction of long‐term potentiation at perforant path synapses of DG engram cells restores both spine density and long‐term memory. This suggests that structural modifications at the level of dendritic spines are a key mechanism for rescuing memory function and that enhancing synaptic connectivity within memory‐relevant neuronal ensembles can effectively counteract deficits associated with early neurodegeneration.

While our findings strongly support the idea that impaired reactivation of learning‐tagged LEC ensembles underlies the retrieval deficit, we cannot exclude the possibility that deficits in consolidation also contribute to the memory impairment in APP mice. Indeed, structural plasticity, such as increases in spine density, has been proposed to reflect consolidation‐related processes that stabilize memory traces.^43^ In line with this, our chemogenetic intervention not only restored retrieval performance but also promoted spine changes on learning‐activated LEC neurons, suggesting that the manipulation may have alleviated consolidation‐related deficits as well.

Thus, memory impairment in APP mice may arise from an interplay between impaired consolidation‐dependent plasticity and insufficient reactivation of memory‐encoding ensembles during memory recall. The alteration of spine density and morphology observed in APP mice may be related to early microglial activation. Indeed, reactive astrocytes and microglia are detectable in the hippocampus by 2 to 3 months of age^44^; moreover, microglial signaling has been implicated in complement‐dependent synapse loss^45^ and RAGE‐p38MAPK‐mediated plasticity deficits in the EC.^17^ While these findings suggest a contribution of microglial activity to synaptic alterations, whether the defect in ensemble reactivation that we report is directly associated with microglial activation remains to be determined.

As AD pathology progresses, hippocampal dysfunction emerges,^11^, ^43^ leading to additional cognitive deficits, including impairments in non‐associative recognition memory, such as those observed in the novel ORT.^17^ We found that in 6‐month‐old APP mice, basal c‐Fos staining in the hippocampus was abnormally elevated and unresponsive to behavioral stimuli. While the total number of tagged neurons does not differ between WT and APP mice, the lack of response to behavioral activation suggests that in APP mice, part of the tagged neurons are not stimulus‐related but were already active at the time of stimulation, leading to reduced ensemble sparsity that may contribute to memory impairment. In addition, reduced sparsity may compromise the specificity of the memory ensemble and increase interference from non‐relevant neuronal populations, disrupting engram‐specific memory retrieval.^46^ This suggests a compromised ability to modulate hippocampal c‐Fos expression in response to environmental cues, potentially reflecting dysregulated network activity. The elevated basal c‐Fos levels in the hippocampus of 6‐month‐old APP mice may reflect Aβ‐driven disruptions in excitatory‐inhibitory balance. It has been shown that 6‐month‐old APP mice exhibit aberrant excitatory activity, leading to epileptiform discharges, compensatory inhibitory remodeling, and synaptic plasticity deficits, particularly in the DG.^28^ This maladaptive network activity may alter the dynamic regulation of c‐Fos expression in response to behavioral stimuli. The altered network activity does not appear to completely prevent the learning of information. However, it significantly impairs the ability to retrieve stored memories using natural cues. Supporting this idea, we found that chemogenetic reactivation of DG learning‐tagged neurons restores novel object recognition memory in 6‐month‐old APP mice. It is important to note that the natural cue‐induced reactivation of learning‐tagged neuronal subpopulations in the DG is significantly reduced in APP mice compared to WT controls. This suggests that elevated basal c‐Fos in the DG may affect the functional recruitment of engram cells, thereby disrupting the precise reactivation of memory‐specific neural ensembles. Regarding possible sex‐related differences, the statistical analysis (Table S1–S10) revealed no significant effect in any of the behavioral or morphological measures, indicating that our results are consistent across sexes.

In conclusion, our findings contribute to the understanding of progressive memory impairment in the APP mouse model. We demonstrate that at an early stage, deficits are characterized by a selective dysfunction in the LEC, specifically in the reactivation of learning‐tagged neurons, which impairs memory retrieval. In contrast, at later stages, behavioral deficits also emerge in less cognitively demanding tasks, indicating a progression of neuronal impairment along the entorhinal‐hippocampal network. Chemogenetic reactivation of neuronal ensembles in the LEC and DG was sufficient to restore memory performance, emphasizing the potential of targeting these circuits to mitigate memory deficits in AD.

## CONFLICT OF INTEREST STATEMENT

No potential conflicts of interest were reported by the authors. Author disclosures are available in the Supporting Information.

## CONSENT STATEMENT

No human subjects were part of this research.

## Supporting information



Supporting information

Supporting information

Supporting information
