# Supplementary material for: Chemogenetic manipulation of learning‐tagged neurons is sufficient to rescue progressive memory deficits in a mouse model of Alzheimer's disease
Source: Alzheimers Dement. 2025 Nov 27;21(11):e70953. doi: 10.1002/alz.70953 (PMC12658379; doi:10.1002/alz.70953)
Supplement: Supplementary file 1 — Supporting information [file ALZ-21-e70953-s003.pdf]

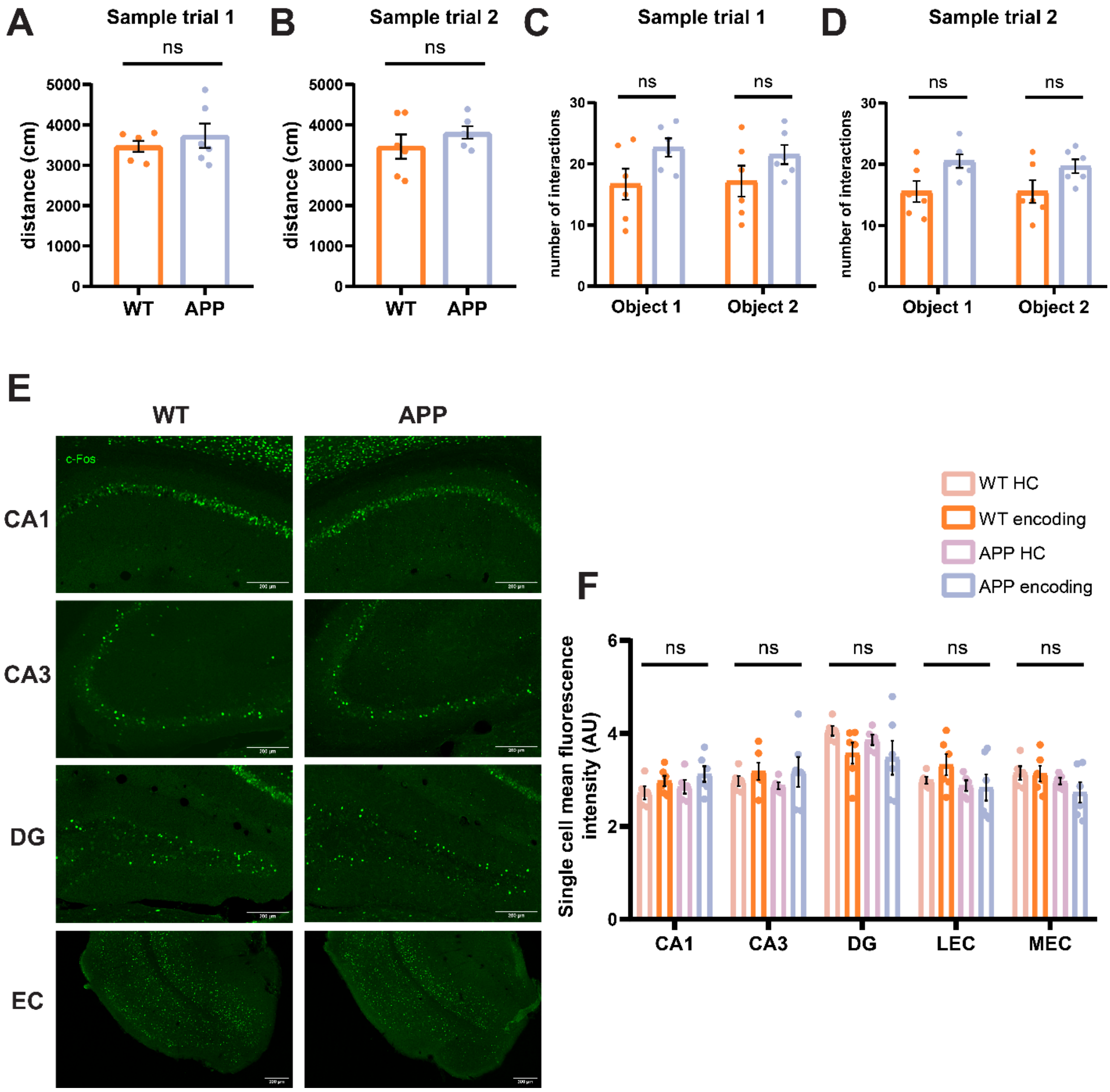

Figure S1. Behavioral analysis and c-Fos quantification showed no alterations during object-place-context memory encoding in 2-month-old APP mice.

(A) No significant difference in the distance travelled during Sample trial 1 (WT 3470 ± 136.7 vs APP 3732 ± 301.8, ns p = 0.4469, df = 10, t = 0.7917, two-tailed unpaired t test).

(B) No significant difference in the distance travelled during Sample trial 2 (WT 3460 ± 299.5 vs APP 3805 ± 154.7, ns p = 0.3302, df = 10, t = 1.023, two-tailed unpaired t test).

(C) No significant difference in the number of interactions with objects during Sample trial 1 between groups (two-way ANOVA with Sidak’s multiple comparisons test: WT Object 1

16.67 ± 2.512 n = 6 vs APP Object 1 22.67 ± 1.498 n = 6, ns p = 0.1073; WT Object 2 17.17 ± 2.535 n = 6 vs APP Object 2 21.5 ± 1.544 n = 6, ns p = 0.2893).

(D) No significant difference in the number of interactions with objects during Sample trial 2 between groups (two-way ANOVA with Sidak’s multiple comparisons test: WT Object 1 15.50 ± 1.727 n = 6 vs APP Object 1 20.50 ± 1.118 n = 6, ns p = 0.0655; WT Object 2 15.50 ± 1.857 n = 6 vs APP Object 2 19.67 ± 1.116 n = 6, ns p = 0.1213).

(E) Representative images showing c-Fos expression (green) in the regions of interest for both groups in the home-cage control condition.

(F) No significant difference in c-Fos$^+$ relative intensity was observed across all regions of interest between the home-cage (HC) and encoding conditions in both WT and APP groups (two-way ANOVA with Sidak’s multiple comparisons test: WT HC CA1 2.722 ± 0.139 n = 5 vs WT encoding CA1 2.976 ± 0.113 n = 6, ns p > 0.9999; WT HC CA3 2.975 ± 0.11 n = 5 vs WT encoding CA3 3.182 ± 0.184 n = 6, ns p > 0.9999; WT HC DG 4.055 ± 0.10 n = 5 vs WT encoding DG 3.574 ± 0.225 n = 6, ns p = 0.9447; WT HC LEC 2.990 ± 0.076 n = 5 vs WT encoding LEC 3.327 ± 0.224 n = 6, ns p = 0.9997; WT HC MEC 3.145 ± 0.146 n = 5 vs WT encoding MEC 3.132 ± 0.164 n = 6, ns p > 0.9999; APP HC CA1 2.851 ± 0.145 n = 5 vs APP encoding CA1 3.123 ± 0.167 n = 6, ns p > 0.9999; APP HC CA3 2.876 ± 0.072 n = 5 vs APP encoding CA3 3.171 ± 0.318 n = 6, ns p > 0.9999; APP HC DG 3.857 ± 0.111 n = 5 vs APP encoding DG 3.477 ± 0.364 n = 6, ns p = 0.9975; APP HC LEC 2.874 ± 0.115 n = 5 vs APP encoding LEC 2.835 ± 0.282 n = 6, ns p > 0.9999; APP HC MEC 2.972 ± 0.07 n = 5 vs APP encoding MEC 2.732 ± 0.223 n = 6, ns p > 0.9999). Data are presented as mean ± SEM.

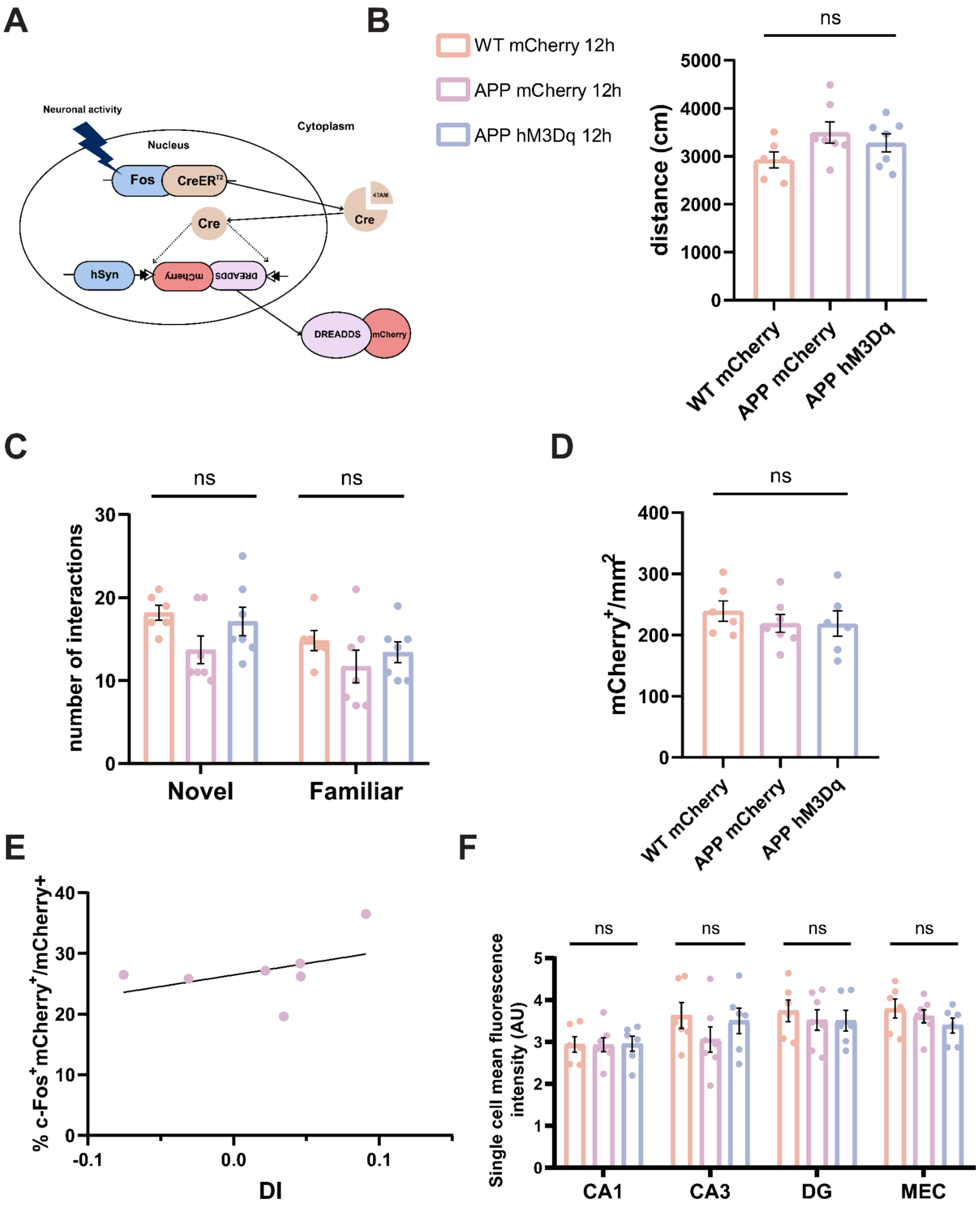

Figure S2. Chemogenetic reactivation of lateral entorhinal cortex learning-tagged neurons restores Object-Place-Context memory in 2-month-old APP mice.

(A) The TRAP strategy allows activity-dependent genetic labeling of neurons. In neurons expressing c-Fos, the TRAP system enables the transient expression of CreERT2. Upon tamoxifen administration, CreERT2 translocates to the nucleus and mediates recombination of a loxP-flanked sequence, inducing permanent expression of the selected protein.

(B) Distance analysis confirmed that chemogenetic manipulation did not affect the motor behavior of APP hM3Dq mice during the recall phase (one-way ANOVA with Sidak's multiple comparisons test: WT mCherry 2926 ± 166.1 n=6 vs APP mCherry 3495 ± 223.5 n=7, ns p = 0.1692; WT mCherry 2926 ± 166.1 n=6 vs APP hM3Dq 3280 ± 185.9 n=7, ns p = 0.5388; APP mCherry vs APP hM3Dq, ns p = 0.8223).

(C) Chemogenetic manipulation did not alter the number of interactions with the object during the recall phase (two-way ANOVA with Sidak's multiple comparisons test: WT mCherry Novel 18.17 ± 0.90 n = 6 vs APP mCherry Novel 13.71 ± 1.658 n = 7, ns p = 0.1470; WT mCherry Novel vs APP hM3Dq Novel 17.14 ± 1.71 n = 7, ns p = 0.9555; APP mCherry Novel vs APP hM3Dq Novel, ns p = 0.3072; WT mCherry Familiar 14.83 ± 1.195 n = 6 vs APP mCherry Familiar 11.71 ± 1.973 n = 7, ns p = 0.4213; WT mCherry Familiar vs APP hM3Dq Familiar 13.43 ± 1.251 n = 7, ns p = 0.8953; APP mCherry Familiar vs APP hM3Dq Familiar, ns p = 0.8074).

(D) No significant differences in the density of LEC tagged neurons were observed between the three groups (one-way ANOVA with Sidak's multiple comparisons test: WT mCherry 239.4 ± 16.58 n = 4 vs APP mCherry 219 ± 14.56 n = 6, ns p = 0.7940; WT mCherry vs APP hM3Dq 218.5 ± 20.61 n = 6, ns p = 0.7995; APP mCherry vs APP hM3Dq, ns p > 0.9999).

(E) No significant correlation was observed between the percentage of c-Fos^+ $mCherry^+$ overlap and the DI for the APP mCherry control group (Pearson correlation, p = 0.3483, correlation coefficient = 0.1763).

(F) No significant differences were observed in the mean fluorescence intensity of individual cells within the hippocampus or the MEC (two-way ANOVA with Sidak’s multiple comparisons test: WT mCherry CA1 2.94 ± 0.183 n = 6 vs APP mCherry CA1 2.933 ± 0.168 n = 7, ns p = 0.9998; WT mCherry CA1 vs APP hM3Dq CA1 2.958 ± 0.181 n = 7, ns p = 0.9984; APP mCherry CA1 vs APP mCherry CA1, ns p = 0.9968; WT mCherry CA3 3.633 ± 0.307 n = 6 vs APP mCherry CA3 3.057 ± 0.302 n = 7, ns p = 0.1985; WT mCherry CA3 vs APP hM3Dq CA3 3.502 ± 0.307 n = 7, ns p = 0.9228; APP mCherry CA3 vs APP mCherry CA3 ns p = 0.3767; WT mCherry DG 3.742 ± 0.261 n = 6 vs APP mCherry DG 3.523 ± 0.243 n = 7, ns p = 0.7872; WT mCherry DG vs APP hM3Dq DG 3.506 ± 0.246 n = 7, ns p = 0.7729; APP mCherry DG vs APP mCherry DG, ns p = 0.9986; WT mCherry MEC 3.797 ± 0.228 n = 6 vs APP mCherry MEC 3.612 ± 0.158 n = 7, ns p = 0.8426; WT mCherry MEC vs APP hM3Dq MEC 3.394 ± 0.177 n = 7, ns p = 0.4729; APP mCherry MEC vs APP mCherry MEC, ns p = 0.7873) Data are presented as mean ± SEM.

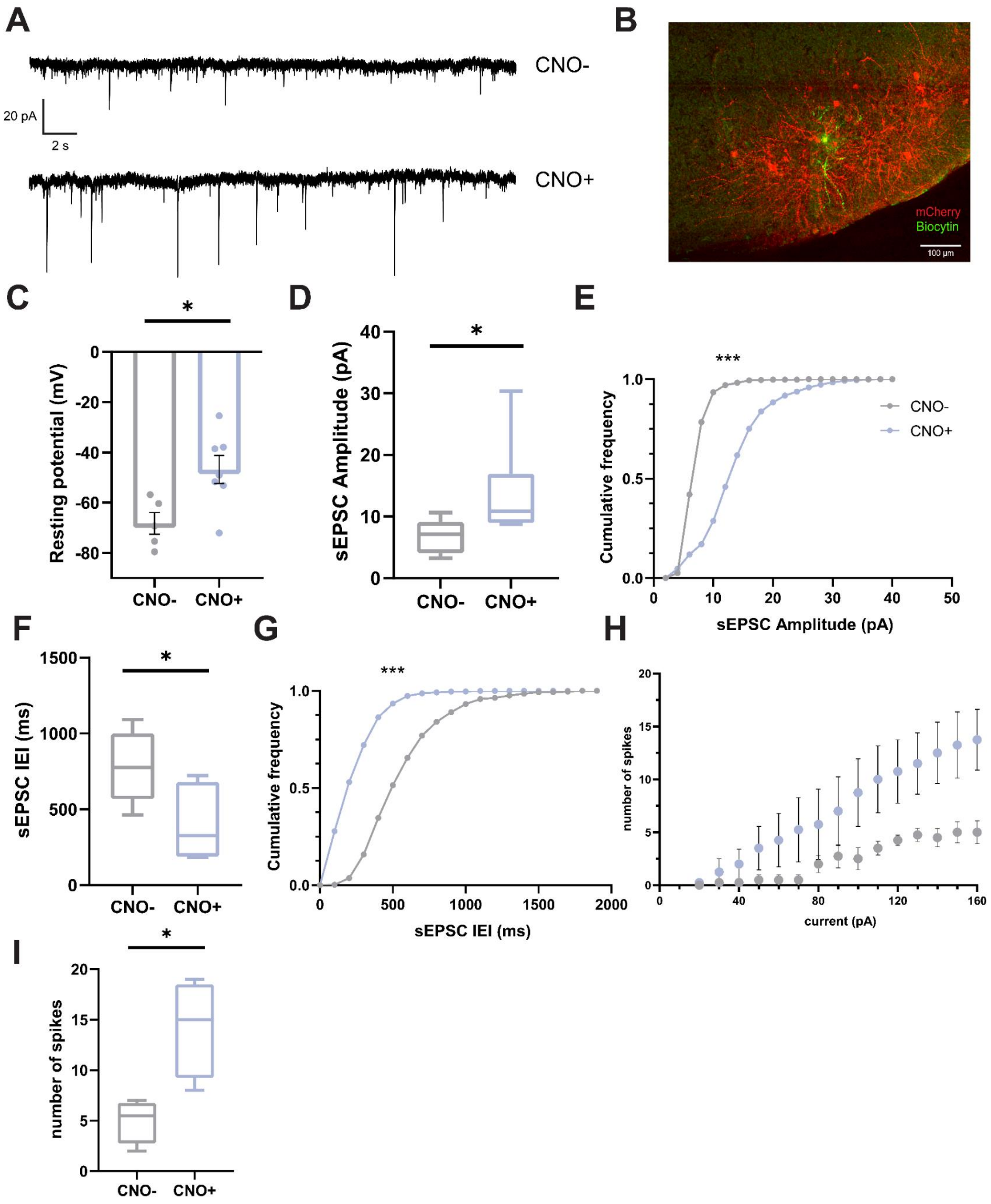

Figure S3. Chemogenetic activation increases excitability of LEC hM3Dq-tagged neurons.

(A) Representative traces of spontaneous excitatory postsynaptic currents (sEPSCs) recorded from superficial layer LEC neurons in slices obtained from APP mice with or without CNO treatment.

(B) Example of a recorded neuron labeled with mCherry and biocytin (mCherry red, biocytin green).

(C) Neurons treated with CNO exhibited a higher resting membrane potential compared to CNO- neurons (two-tailed unpaired t test : CNO- -68.25 ± 4.311 n = 5 vs CNO+ -46.81 ± 5.580 n = 6, * p = 0.0180, df = 10, t = 2.825).

(D) Neurons treated with CNO exhibited a higher peak amplitude compared to CNO- neurons (Mann-Whitney test: CNO- 6.688 ± 1.262 n = 5 vs CNO+ 13.71 ± 3.372 n = 6, * p = 0.03, U = 3).

(E) Cumulative frequency curves showed that sEPSC peak amplitudes were significantly larger in CNO+ neurons than in CNO- neurons (Kolmogorov-Smirnov test: *** $p < 0.0001$, D = 0.6671).

(F) The inter-event interval (IEI) of sEPSCs was significantly shorter in CNO+ neurons relative to controls (Mann-Whitney test: CNO- 782.4 ± 106.1 n = 5 vs CNO+ 401.9 ± 98.06 n = 6, * p = 0.03, U = 3).

(G) Cumulative frequency analysis revealed a leftward shift in the distribution of inter-event intervals in CNO-treated neurons (Kolmogorov-Smirnov test: *** $p < 0.0001$, D = 0.5749).

(H) Number of action potentials per current step showed a tendency to increase in CNO+ neurons compared to CNO- neurons (two-way repeated-measures ANOVA: interaction $F(14, 84) = 3.945$, $p < 0.0001$; current step $F(14, 84) = 23.18$, $p < 0.0001$; treatment factor $F(1, 6) = 3.926$, $p = 0.0948$; subject effect $F(6, 84) = 50.93$, $p < 0.0001$).

(I) The number of action potentials evoked by the maximal current step (160 pA) was significantly higher in CNO+ neurons than in controls (Mann-Whitney test: CNO- 5 ± 1.08 n = 4 vs CNO+ 14.25 ± 2.428 n = 4, * p = 0.0286, U = 0). Each data point represents a

single recorded cell; n refers to the total number of patched neurons obtained from 3 APP mice (2 m.o.). Data are presented as mean ± SEM.

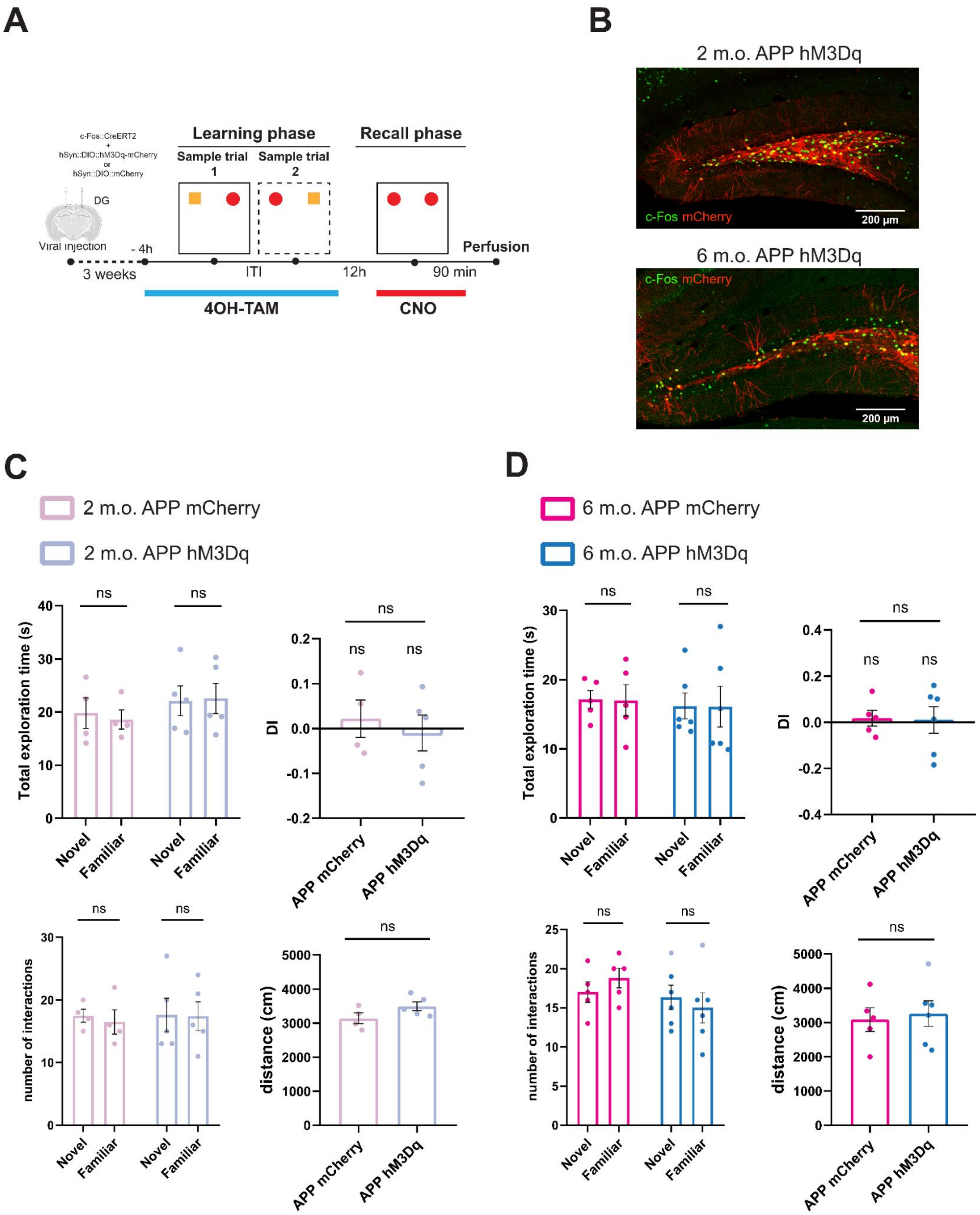

Figure S4. Chemogenetic reactivation of dentate gyrus learning-tagged neurons failed to restore Object-Place-Context memory in 2-month-old and 6-month-old APP mice.

(A) Schematic representation of the OPCRT: Viral injections were performed in the dentate gyrus 3 weeks before the start of behavioral procedures.4OH-TAM treatment was administered 4 hours prior to the learning phase, while CNO injection was given 30 minutes before the recall phase. Animals were perfused 90 minutes after the recall phase.

(B) Representative images of mCherry (red) and c-Fos (green) expressing neurons in the DG of 2m.o. and 6 m.o. APP hM3Dq groups. Scale bars are shown in the figures.

(C) Chemogenetic manipulation of DG learning-tagged ensembles in 2 m.o. APP mice failed to rescue memory performance (Time exploration two-way ANOVA with Sidak's multiple comparisons test: APP mCherry Novel 19.80 ± 2.88 n = 4 vs APP mCherry Familiar 18.60 ± 1.83 n = 4, ns p = 0.9482; APP hM3Dq Novel 22.10 ± 2.80 n = 5 vs APP hM3Dq Familiar 22.58 ± 2.85 n = 5, ns p = 0.9891; DI one-sample t test: APP mCherry 0.021 ± 0.041, n = 4, ns p = 0.6383, df = 3, t = 0.5211; APP hM3Dq -0.01 ± 0.04, n = 5, ns p = 0.8116, df = 4, t = 0.2546; DI two-tailed unpaired t test : APP mCherry vs APP hM3Dq, ns p = 0.6011, df = 7, t = 0.5474; interactions two-way ANOVA with Sidak's multiple comparisons test: APP mCherry Novel 17.50 ± 1.04 n = 4 vs APP mCherry Familiar 16.50 ± 1.93 n = 4, ns p = 0.9457; APP hM3Dq Novel 17.60 ± 2.67 n = 5 vs APP hM3Dq Familiar 17.40 ± 2.29 n = 5, ns p = 0.9972; distance two-tailed unpaired t test : APP mCherry 3142 ± 159.3, n = 4 vs APP hM3Dq 3493 ± 129.6, n = 5, ns p = 0.1272, df = 7, t = 1.730).

(D) Chemogenetic manipulation of DG learning-tagged ensembles in 6 m.o. APP mice failed to restore OPC memory performance (Time exploration two-way ANOVA with Sidak's multiple comparisons test: APP mCherry Novel 17.15 ± 1.26 n = 5 vs APP mCherry Familiar 17.01 ± 2.27 n = 5, ns p = 0.9989; APP hM3Dq Novel 16.19.10 ± 1.86 n = 6 vs APP hM3Dq Familiar 16.11 ± 2.94 n = 6, ns p = 0.9995; DI one-sample t test: APP mCherry 0.018 ± 0.034, n = 4, ns p = 0.6252, df = 4, t = 0.5284; APP hM3Dq 0.009 ± 0.058, n = 6, ns p = 0.8723, df = 5, t = 0.1692; DI two-tailed unpaired t test : APP mCherry

vs APP hM3Dq, ns p = 0.9112, df = 9, t = 0.1147; interactions two-way ANOVA with Sidak's multiple comparisons test: APP mCherry Novel 17 ± 1.30 n = 5 vs APP mCherry Familiar 18.80 ± 1.24 n = 5, ns p = 0.7011; APP hM3Dq Novel 16.33 ± 1.54 n = 6 vs APP hM3Dq Familiar 15 ± 1.93 n = 6, ns p = 0.79; distance two-tailed unpaired t test : APP mCherry 3083 ± 344.4, n = 5 vs APP hM3Dq 3260 ± 374.4, n = 6, ns p = 0.7396, df = 9, t = 0.3429).

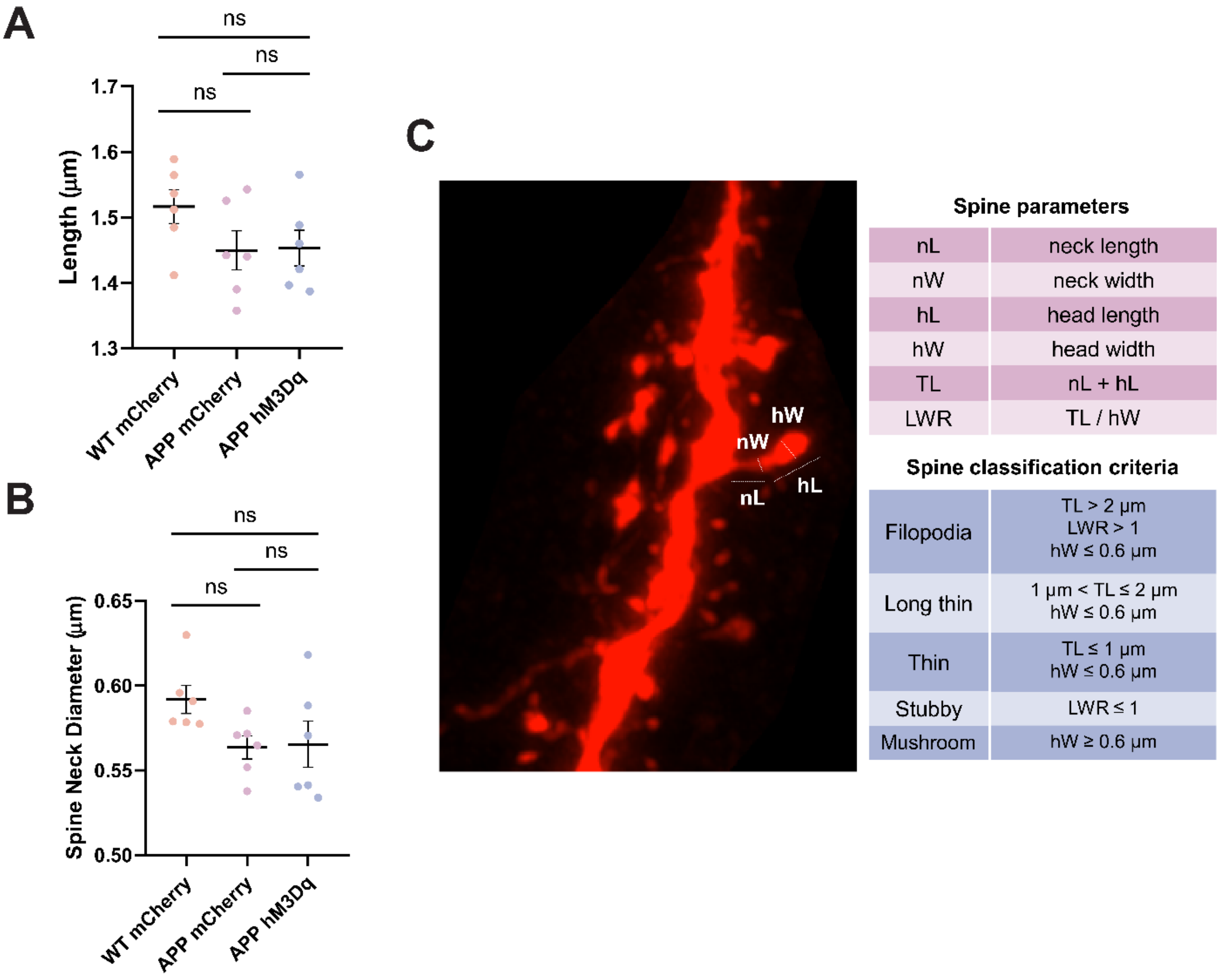

Figure S5. Chemogenetic reactivation of learning-tagged neurons promotes a shift in dendritic spine morphology

(A) No significant difference in spine length was detected between groups (one-way ANOVA with Sidak's multiple comparisons test: WT mCherry 1.516 ± 0.025 n = 6 vs APP mCherry 1.450 ± 0.029 n = 6, ns p = 0.2943; WT mCherry vs APP hM3Dq 1.453 ± 0.02, ns p = 0.3327; APP mCherry vs APP hM3Dq, ns p = 0.9997).

(B) No significant difference in spine neck diameter was observed between groups (one-way ANOVA with Sidak's multiple comparisons test: WT mCherry 0.591 ± 0.008 n = 6 vs APP mCherry 0.563 ± 0.006 n = 6, ns p = 0.1791; WT mCherry vs APP hM3Dq 0.565 ± 0.013, ns p = 0.2215; APP mCherry vs APP hM3Dq, ns p = 0.9991).

(C) Representative image of a dendrite from the analyzed dataset, outlined to highlight the parameters used in the morphological analysis. Key features of the dendritic spines are summarized in the Spine Parameters table (here, TL is the Total Length of the spine, and LWR is the Length-to-Width ratio). The rules used for classification based on the morphology parameters are summarized in the Spine Classification Criteria table. Spine morphological features are presented in µm. Data are presented as mean ± SEM.

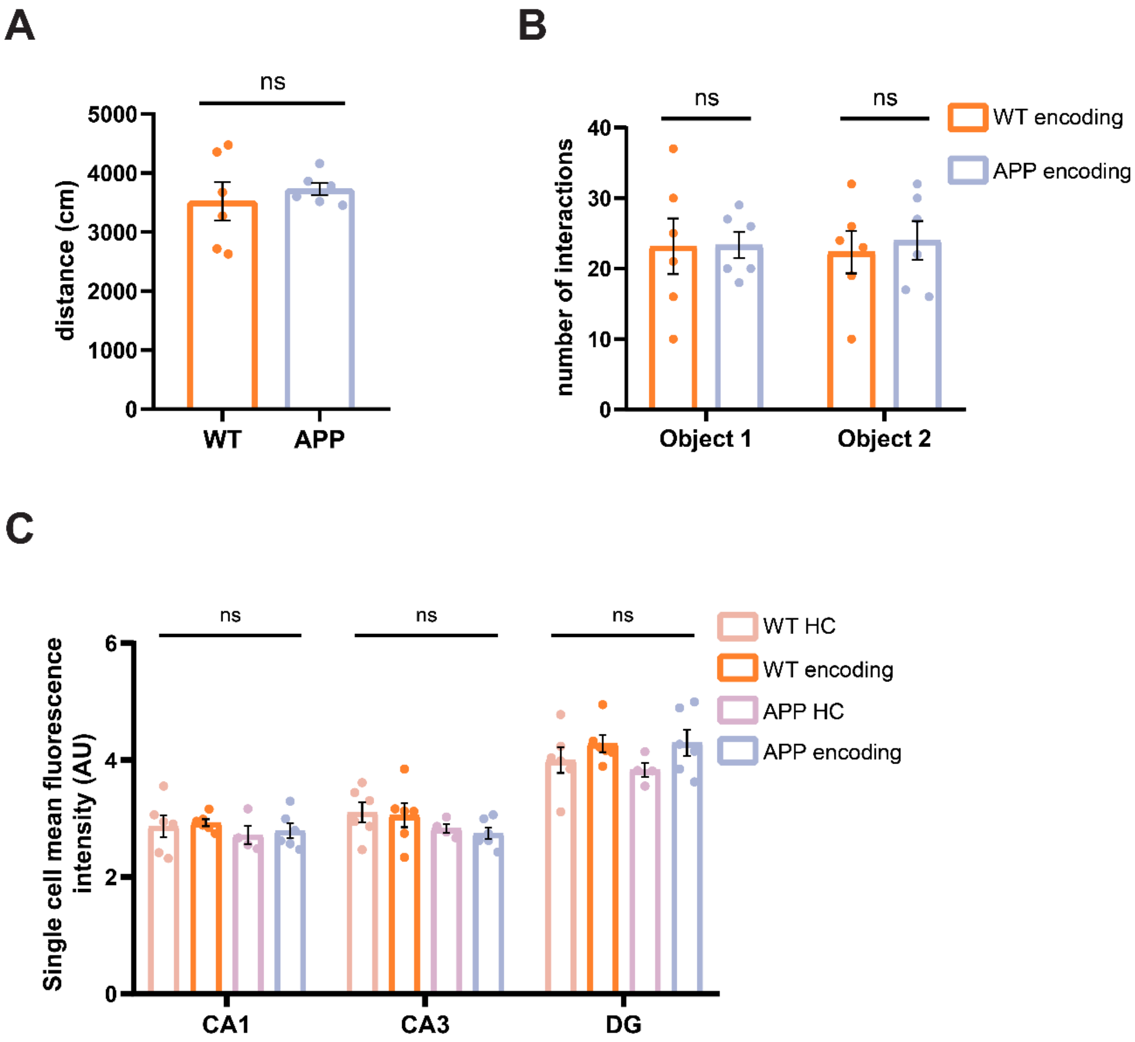

Figure S6. Impaired hippocampal c-Fos activation following behavioral stimulation in 6-month-old APP mice

(A) Distance analysis showed no alteration of motor behavior in 6-month-old APP mice compared to the WT control group (WT encoding 3515 ± 323.1 n = 6 vs APP encoding 3728 ± 106.6 n = 6, ns p = 0.5527, df = 10, t = 0.6143, two-tailed unpaired t test).

(B) Analysis of the number of interactions confirmed that exploratory behavior in 6-month-old APP mice was not altered (two-way ANOVA with Sidak's multiple comparisons test: WT encoding Object 1 23.17 ± 3.962 n = 6 vs APP encoding Object 1 23.33 ± 1.856 n = 6, ns p = 0.9990; WT encoding Object 2 22.33 ± 3.018 n = 6 vs APP encoding Object 2 24 ± 2.745 n =6, ns p = 0.9086).

(C) No significant difference in c-Fos$^+$ relative intensity was observed across all regions of interest between the home-cage (HC) and encoding conditions in both WT and APP groups (two-way ANOVA with Sidak's multiple comparisons test: WT HC CA1 2.868 ± 0.185 n = 6 vs WT encoding CA1 2.928 ± 0.057 n = 6, ns p > 0.9999; WT HC CA1 vs APP HC CA1 2.718 ± 0.153 n = 4, ns p > 0.9999; WT HC CA1 vs APP encoding CA1 2.791 ± 0.126 n = 6, ns p > 0.9999; WT encoding CA1 vs APP encoding CA1, ns p > 0.9999; APP HC CA1 vs APP encoding CA1, ns p > 0.9999; WT HC CA3 3.107 ± 0.173 n = 6 vs WT encoding CA3 3.107 ± 0.173 n = 6, ns p > 0.9999; WT HC CA3 vs APP HC CA3 2.83 ± 0.076 n = 4, ns p > 0.9999; WT HC CA3 vs APP encoding CA3 2.746 ± 0.099 n = 6, ns p = 0.9970; WT encoding CA3 vs APP encoding CA3, ns p = 0.8883; APP HC CA3 vs APP encoding CA3, ns p = 0.9674; WT HC DG 4 ± 0.221 n = 6 vs WT encoding DG 4.282 ± 0.145 n = 6, ns p = 0.9874; WT HC DG vs APP HC DG 3.833 ± 0.12 n = 4, ns p > 0.9999; WT HC DG vs APP encoding DG 4.296 ± 0.224 n = 6, ns p = 0.9794; WT encoding DG vs APP encoding DG, ns p > 0.9999; APP HC DG vs APP encoding DG, ns p = 0.7380) Data are presented as mean ± SEM.

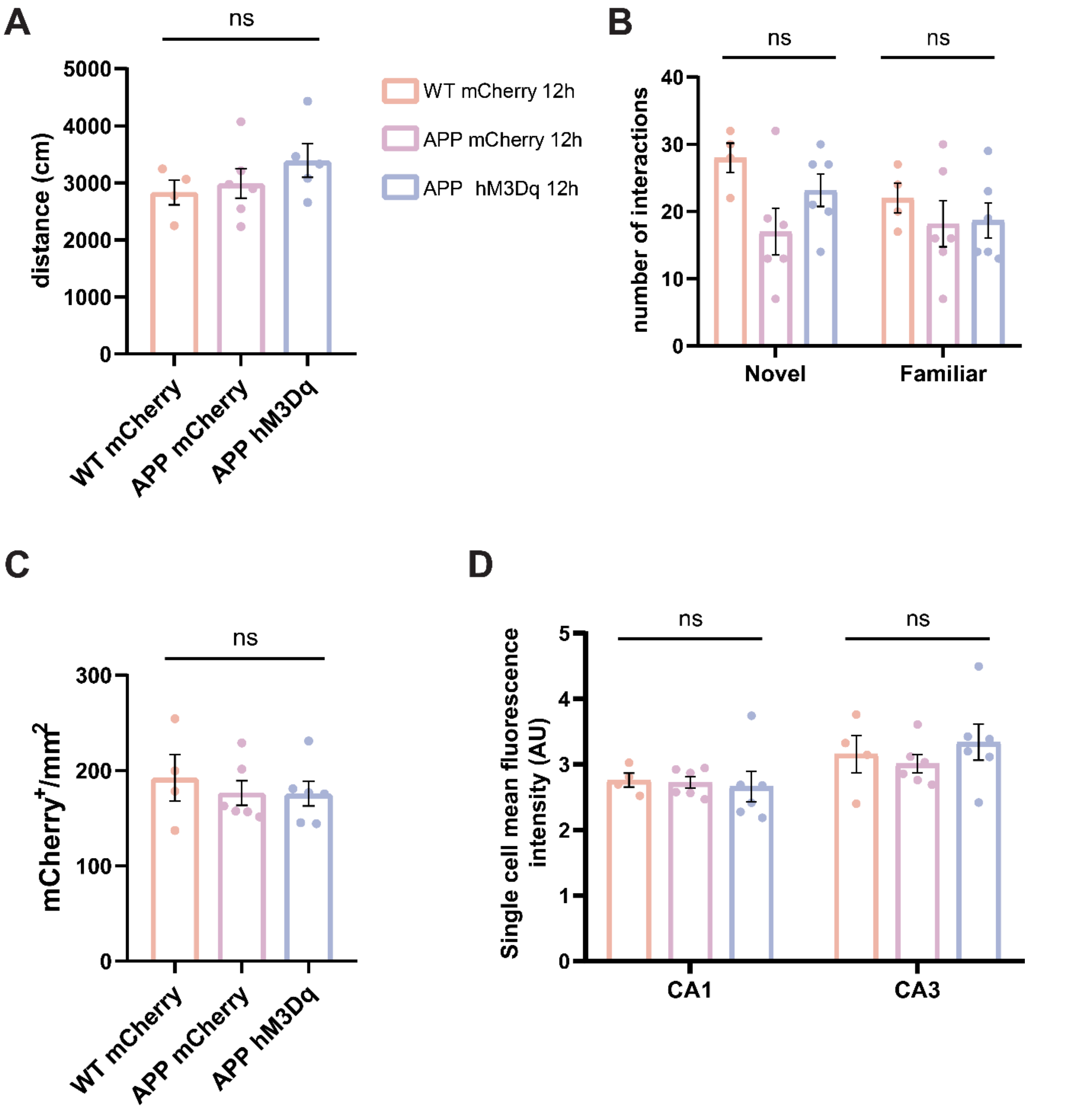

Figure S7. Chemogenetic reactivation of dentate gyrus learning-tagged neurons rescues Novel Object Recognition in 6-month-old APP mice.

(A) No significant differences in motor activity were observed during the recall phase between the three groups (one-way ANOVA with Sidak's multiple comparisons test: WT mCherry 2833 ± 217.1 n = 4 vs APP mCherry 2989 ± 258.2 n = 6, ns p = 0.9714; WT mCherry vs APP hM3Dq 3390 ± 293.7 n = 6, ns p = 0.4692; APP mCherry vs APP hM3Dq, ns p = 0.6412).

(B) There were no significant differences in the number of interactions with the objects during the recall phase (two-way ANOVA with Sidak's multiple comparisons test: WT mCherry Novel 28 ± 2.160 n = 4 vs APP mCherry Novel 17 ± 3.474 n = 6, ns p = 0.0557; WT mCherry Novel vs APP hM3Dq Novel 23.17 ± 2.414 n= 6, ns p = 0.6290; APP mCherry Novel vs APP hM3Dq Novel, ns p = 0.3387;

WT mCherry Familiar 22 ± 2.198 n = 4 vs APP mCherry Familiar 18.17 ± 3.429 n = 6, ns p = 7742; WT mCherry Familiar vs APP hM3Dq Familiar 18.67 ± 2.591 n = 6, ns p = 0.8381; APP mCherry Familiar vs APP hM3Dq Familiar, ns p = 0.9990).

(C) No significant differences in the density of DG tagged neurons were observed between the three groups (one-way ANOVA with Sidak's multiple comparisons test: WT mCherry 192.4 ± 24.38 n = 4 vs APP mCherry 176.4 ± 12.85 n = 6, ns p = 0.8793; WT mCherry vs APP hM3Dq 175.6 ± 12.88 n = 6, ns p = 0.8633; APP mCherry vs APP hM3Dq, ns p > 0.9999).

(D) No significant differences were observed in the mean relative fluorescence intensity within CA1 and CA3 (two-way ANOVA with Sidak's multiple comparisons test: WT mCherry CA1 2.762 ± 0.105 n = 4 vs APP mCherry CA1 2.725 ± 0.086 n = 6, ns p = 0.9919; WT mCherry CA1 vs APP hM3Dq CA1 2.665 ± 0.230 n = 6, ns p = 0.9456; APP mCherry CA1 vs APP mCherry CA1, ns p = 0.9736; WT mCherry CA3 3.158 ± 0.283 n = 4 vs APP mCherry CA3 3.011 ± 0.136 n = 6, ns p = 0.8788; WT mCherry CA3 vs APP hM3Dq CA3 3.339 ± 0.274 n = 6, ns p = 0.8233; APP mCherry CA3 vs APP mCherry CA3 ns p = 0.4583). Data are presented as mean ± SEM.
